# Clinical and genetic characteristics of 251 consecutive patients with macular and cone/cone-rod dystrophy

**DOI:** 10.1038/s41598-018-22096-0

**Published:** 2018-03-19

**Authors:** Johannes Birtel, Tobias Eisenberger, Martin Gliem, Philipp L. Müller, Philipp Herrmann, Christian Betz, Diana Zahnleiter, Christine Neuhaus, Steffen Lenzner, Frank G. Holz, Elisabeth Mangold, Hanno J. Bolz, Peter Charbel Issa

**Affiliations:** 1 Department of Ophthalmology, University of Bonn, Bonn, Germany; 2 Center for Rare Diseases Bonn (ZSEB), University of Bonn, Bonn, Germany; 3 Bioscientia Center for Human Genetics, Ingelheim, Germany; 4 Institute of Human Genetics, University of Bonn, Bonn, Germany; 5 Institute of Human Genetics, University Hospital of Cologne, Cologne, Germany; 6 Oxford Eye Hospital, Oxford University Hospitals NHS Foundation Trust, and Nuffield Laboratory of Ophthalmology, Department of Clinical Neurosciences, University of Oxford, Oxford, UK

## Abstract

Macular and cone/cone-rod dystrophies (MD/CCRD) demonstrate a broad genetic and phenotypic heterogeneity, with retinal alterations solely or predominantly involving the central retina. Targeted next-generation sequencing (NGS) is an efficient diagnostic tool for identifying mutations in patient with retinitis pigmentosa, which shows similar genetic heterogeneity. To detect the genetic causes of disease in patients with MD/CCRD, we implemented a two-tier procedure consisting of Sanger sequencing and targeted NGS including genes associated with clinically overlapping conditions. Disease-causing mutations were identified in 74% of 251 consecutive MD/CCRD patients (33% of the variants were novel). Mutations in *ABCA4*, *PRPH2* and *BEST1* accounted for 57% of disease cases. Further mutations were identified in *CDHR1, GUCY2D*, *PROM1*, *CRX*, *GUCA1A*, *CERKL*, *MT-TL1*, *KIF11*, *RP1L1*, *MERTK*, *RDH5*, *CDH3*, *C1QTNF5*, *CRB1*, *JAG1*, *DRAM2*, *POC1B*, *NPHP1 and RPGR*. We provide detailed illustrations of rare phenotypes, including autofluorescence and optical coherence tomography imaging. Targeted NGS also identified six potential novel genotype-phenotype correlations for *FAM161A, INPP5E*, *MERTK*, *FBLN5*, *SEMA4A* and *IMPDH1*. Clinical reassessment of genetically unsolved patients revealed subgroups with similar retinal phenotype, indicating a common molecular disease cause in each subgroup.

## Introduction

Macular and cone/cone-rod dystrophies (MD/CCRD) comprise diverse inherited retinal diseases with progressive degeneration, dysfunction and vision loss of the central retina^1^. Characteristic symptoms include decreasing visual acuity, reading difficulties, photophobia and dyschromatopsia. Later, variable loss of rod function with reduced night vision and loss of peripheral visual field may occur^1^. Full-field electroretinography (ffERG) allows classifying patients into those with MD (normal ffERG), CD (only reduced photopic responses) and CRD (reduced photopic and scotopic responses).

Genotype-phenotype correlations of MD/CCRD are often complex. For instance, different mutations in one gene can cause highly diverse phenotypes, and mutations in different genes can cause very similar phenotypes. Furthermore, there is a considerable clinical overlap of CCRD with MD and RP, and the classification may change from MD to CCRD with disease progression^1–3^. Although more than 30 disease-causing genes have been reported for MD/CCRD (RetNet, https://sph.uth.edu/retnet), the genetic disease cause in a substantial number of patients is currently unknown.

As novel therapeutic options including gene therapy are being developed, the identification of the individual mutation has gained importance. Next-generation sequencing (NGS) has become a very efficient diagnostic tool, as exemplified in patients with RP^4–8^. However, only few studies – most of them with limited patient numbers – have reported the performance of targeted NGS for defining the molecular basis of unselected patient cohorts with MD/CCRD^2,9–11^.

Here, we report results from a cohort of 251 unrelated, consecutive and clinically well characterized MD/CCRD patients who underwent extensive molecular genetic analysis. The results provide insights into the diversity of the mutational spectrum of MD/CCRD and indicate potential novel or uncommon genotype-phenotype correlations. Moreover, we reviewed the retinal phenotype of patients in whom the molecular disease cause remained unexplained and propose phenotypic subgroups which may correspond to specific yet unknown genetic disease causes.

## Methods

All methods were carried out in accordance with the approved guidelines. All data generated or analyzed during this study are included in this article (and its Supplementary Information files).

### Patients

This retrospective single-center cross-sectional study included 251 consecutive MD/CCRD patients (235 Caucasian, 139 female) seen between summer 2012 and summer 2015 at the Department of Ophthalmology, University of Bonn, Germany. Inclusion criteria were 1) the clinical diagnosis of a MD/CCRD by one of the senior clinicians (P.C.I., P.H.) based on the patient’s history of visual symptoms, clinical examination, retinal imaging, and – where available – electrophysiology, and 2) that the patient underwent genetic testing for their retinal disease. Patients were excluded if they had an obvious syndromic retinal disease, age-related macular degeneration, central serous retinopathy, autoimmune retinopathy, or retinal vascular disease. Also, patients were excluded if they had the clinical diagnosis of achromatopsia (cone dysfunction syndromes were differentiated from retinal dystrophies, and genotyping was performed in a different lab), X-linked retinoschisis (genetic testing performed in different labs), and North Carolina macular dystrophy (genetic basis not published before 2016). The study was in adherence with the Declaration of Helsinki. Institutional review board approval (Ethics Committee, Medical Faculty, University of Bonn) and patients’ informed consent were obtained. Asymptomatic family members received genetic counselling prior to genetic testing.

A general medical history was obtained from each patient and a pedigree was assembled based on a detailed family history with regards to ocular diseases and visual symptoms. The mode of inheritance was assumed to be autosomal recessive (a.r.) in case of parental consanguinity or if only siblings were affected, autosomal dominant (a.d.) if the family history was suggestive for the same inherited retinal disease in at least 3 successive generations, and X-linked if the disease occurred in different generations without male-to-male transmission and with only males being severely affected while females were normal or with only minor symptoms. If other family members were affected but the pedigree was not suggestive for any of the above patterns, inheritance was classified as inconclusive. Disease in patients without other affected family members and no parental consanguinity was considered as sporadic.

### Image acquisition and functional testing

All patients underwent a standardized clinical examination and retinal imaging, including spectral domain optical coherence tomography (OCT), fundus autofluorescence (AF) imaging (both, Spectralis HRA + OCT, Heidelberg Engineering, Heidelberg, Germany), fundus photography (Zeiss, Visucam, Oberkochen, Germany) and wide-field fundus imaging (Optos PLC, Dunfermline, United Kingdom). To assess retinal function, best corrected visual acuity (BCVA), ffERG, electroocoulogram (EOG) and visual field testing (different devices and protocols depending on availability and patient needs) were performed. In total, ffERG was acquired from 223 patients (89%) and EOG from 48 patients (19%, mainly those with vitelliform lesions).

### Molecular genetic analysis

Genomic DNA was extracted from blood lymphocytes by a standard protocol. A two-tier procedure was implemented to identify the molecular cause of disease: If the retinal phenotype was highly suggestive for mutations in *ABCA4*, *PRPH2* or *BEST1*, Sanger sequencing (and, in case of *ABCA4*, multiplex ligation-dependent probe amplification) for the respective gene was performed. If the result of this initial molecular testing was negative, or if the phenotype was not clearly suggestive for retinopathy associated with these three genes, then 48 genes whose mutations were known to cause MD/CCRD at the time of panel design (2015; *ABCA4*, *ACBD5*, *ADAM9*, *AIPL1*, *BEST1*, *C1QTNF5*, *C21orf2*, *C8orf37*, *CABP4*, *CACNA1F*, *CACNA2D4*, *CDH3*, *CDHR1*, *CERKL*, *CNGA3*, *CNGB3*, *CNNM4*, *CRX*, *CTNNA1*, *ELOVL4*, *GUCA1A*, *GUCA1B*, *GUCY2D*, *IFT140*, *KCNV2*, *MFSD8*, *NR2E3*, *PCYT1A*, *PDE6C*, *PITPNM3*, *POC1B*, *PRDM13*, *PROM1*, *PRPH2*, *RAB28*, *RAX2*, *RBP3*, *RDH5*, *RGS9*, *RGS9BP*, *RIMS1*, *RP1L1*, *RPGR*, *RPGRIP1*, *SEMA4A*, *TIMP3*, *TTLL5*, *UNC119*) were enriched using Roche/NimbleGen sequence capture technology, sequenced on an Illumina HiSeq. 1500 system and bioinformatically evaluated (including analysis for copy number variations) as described previously^8^. To detect X-linked mutations, we added NGS of amplicons comprising *RPGR*_*ORF15*_ (to be described elsewhere) to panel-NGS of the remaining exons of *RPGR*. The NGS panel additionally included all the genes known to be associated with clinically overlapping conditions such as rod-cone dystrophy, Leber’s congenital amaurosis (LCA) and several syndromes with retinal dystrophies. This allowed for an extended genetic assessment if no mutation was found in the “core genes”, without need for additional experimental efforts. Verification of mutations identified in NGS and segregation analyses were carried out by PCR and subsequent Sanger sequencing.

Variants were filtered against dbNSFP v2.0, dbSNP v137, gnomAD (exomes) and the Human Gene Mutation Database (HGMD^®^ Professional 2017.3). The cut-off for the maximum minor allele frequency (MAF) was set to 1%^12^. Nonsense, frameshift, large deletions and canonical splice site variants were regarded pathogenic. Rare non-synonymous single nucleotide variations were considered likely pathogenic when at least half of the algorithms of used in silico prediction software tools predicted that the variant is probably damaging and when it was predicted as conserved with conservation prediction algorithms. The impact of splice site variants was assessed using specific splice site prediction programs.

### Genotype-phenotype correlation

After molecular testing, all patients with identified variants were re-evaluated to survey if their retinal phenotype was compatible with previous descriptions of the retinopathy related to the mutated gene. In case of inconsistency, the respective phenotypes were considered as potential novel genotype-phenotype correlations. Where possible, segregation analysis was performed, although the number of available family members was often small and included mostly the parents and, occasionally, siblings.

## Results

### Mutations in genes known to be involved in MD/CCRD pathogenesis

**Figure 1:**
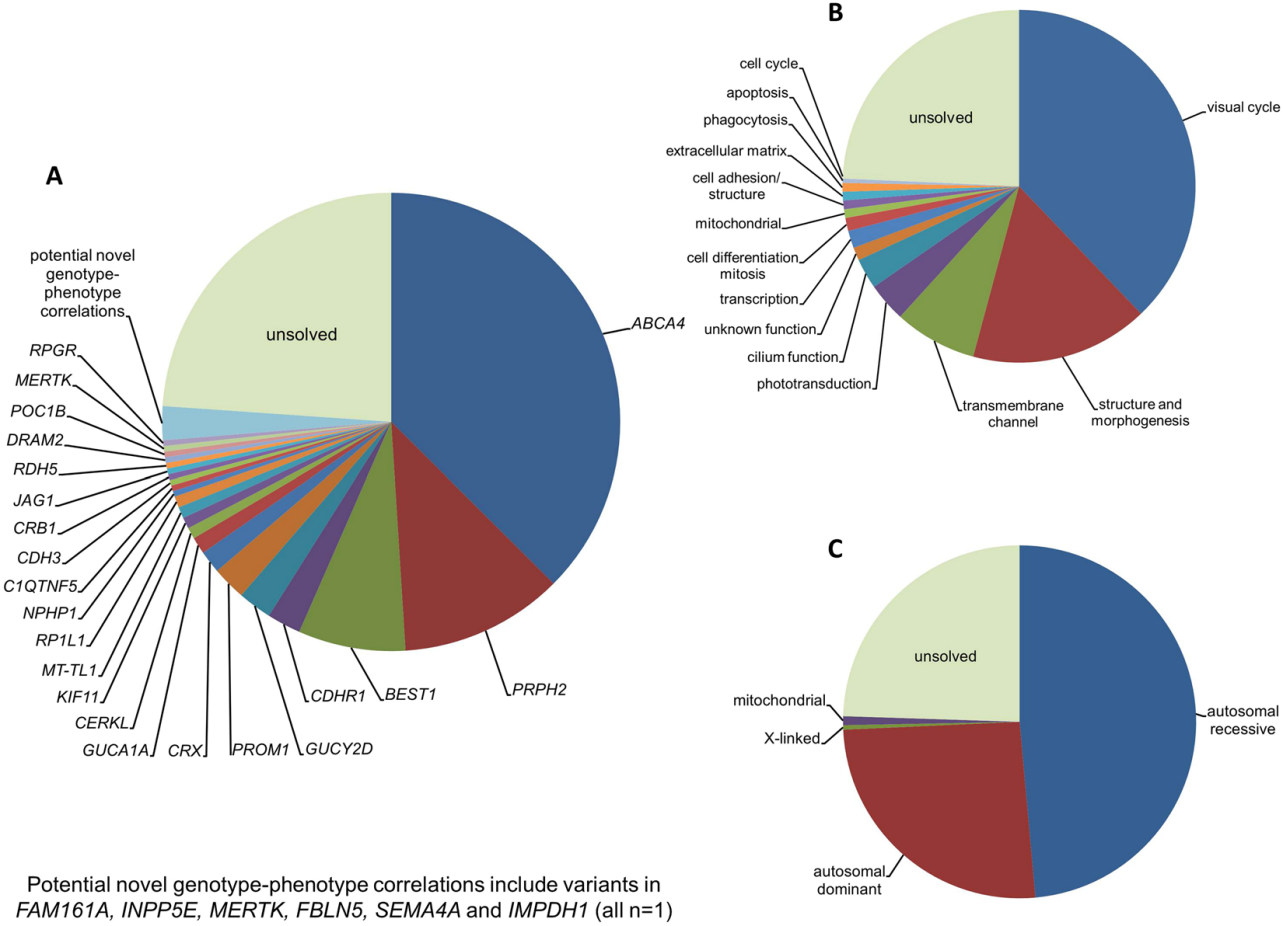
Mutational spectrum (**A**) Spectrum of variants identified in 251 patients affected by macular and cone/cone-rod dystrophies. (**B**) Functional categorization of variants identified in our study. (**C**) Inheritance based on the genetic findings. Percentages refer to patients with mutations in the considered causative gene, pathway or mode of inheritance.

Disease-causing or likely disease-causing mutations were identified in 185 out of 251 patients (74%) with MD/CCRD (Supplementary Table 1). Hereof, 64 (33%) mutations were novel at the time of manuscript submission (Supplementary Table 2). Overall, the 193 different mutations were distributed across 22 genes that encode proteins from diverse pathways and cellular compartments (Fig. 1A,B, Supplementary Table 3).

In 57% of all MD/CCRD cases (77% of the 185 solved cases), retinal disease was explained by mutations in *ABCA4* (n = 94, 37%), *PRPH2* (n = 29, 12%) or *BEST1* (n = 19, 8%; including 5 a.r. and 14 a.d. mutations). Further mutations were identified in *CDHR1* (n = 6), *GUCY2D* (n = 6), *PROM1* (n = 6), *CRX* (n = 4), *GUCA1A* (n = 3), *CERKL* (n = 2), *MT-TL1* (n = 2), *KIF11* (n = 2), *RP1L1* (n = 2), *MERTK* (n = 1), *RDH5* (n = 1), *CDH3* (n = 1), *C1QTNF5* (n = 1), *CRB1* (n = 1), *JAG1* (n = 1), *DRAM2* (n = 1), *POC1B* (n = 1), *NPHP1* (n = 1) and *RPGR* (n = 1) (Fig. 1A, Supplementary Table 1). Although there was considerable phenotypic heterogeneity even between patients with mutations in the same disease-causing gene, the phenotype was compatible with the previously described gene-associated retinal phenotype(s) in all instances. In the 185 solved cases, inheritance based on the genetic findings was autosomal recessive (n = 119, 64%), autosomal dominant (n = 63, 34%), X-linked (n = 1, 1%) and mitochondrial (n = 2, 1%) (Fig. 1C).

If the pedigree fulfilled the criteria for a.r. or a.d. inheritance (see Methods), a molecular diagnosis was achieved in 89% (n = 31 out of 35) or 84% (n = 31 out of 37), respectively, and then confirmed the expected mode of inheritance (Table 1). The pedigree of 3 patients (#54, #63, #83, Supplementary Table 1) with only 2 affected generations was inconclusive, and pseudo-dominant inheritance due to mutations in *ABCA4* explained this finding at the molecular level. The mutation detection rate was lower (68%; n = 120 out of 176) in patients with sporadic MD/CCRD. In these 120 patients, the molecular diagnosis specified inheritance as a.r. in 85 patients (71%), a.d. in 32 (27%) patients, X-linked in 1 (1%) patients and mitochondrial in 2 patients (2%) (Table 1). Out of the 66 unsolved cases, 56 (85%) were sporadic and 10 (15%) had a family history clearly suggestive for a.d. (n = 6) or a.r. (n = 4) inheritance.

**Table 1:** Mode of inheritance based on family history, on genetic results, and mutation detection rate for each group.

Inheritance based on family history	n	Inheritance based on mutation	n	Mutation detection rate %
autosomal recessive	35	autosomal recessive	31	89%
autosomal dominant	37	autosomal dominant	31	84%
X-linked	0	n.a.	0	n.a.
inconclusive	3	autosomal recessive (pseudo-dominant)	3	100%
sporadic	176	autosomal recessive	85	68%
		autosomal dominant	32	
		X-linked	1	
		mitochondrial	2	

### Clinical description of uncommonly observed but characteristic genotype-phenotype correlations

#### *CRB1*

**Figure 2:**
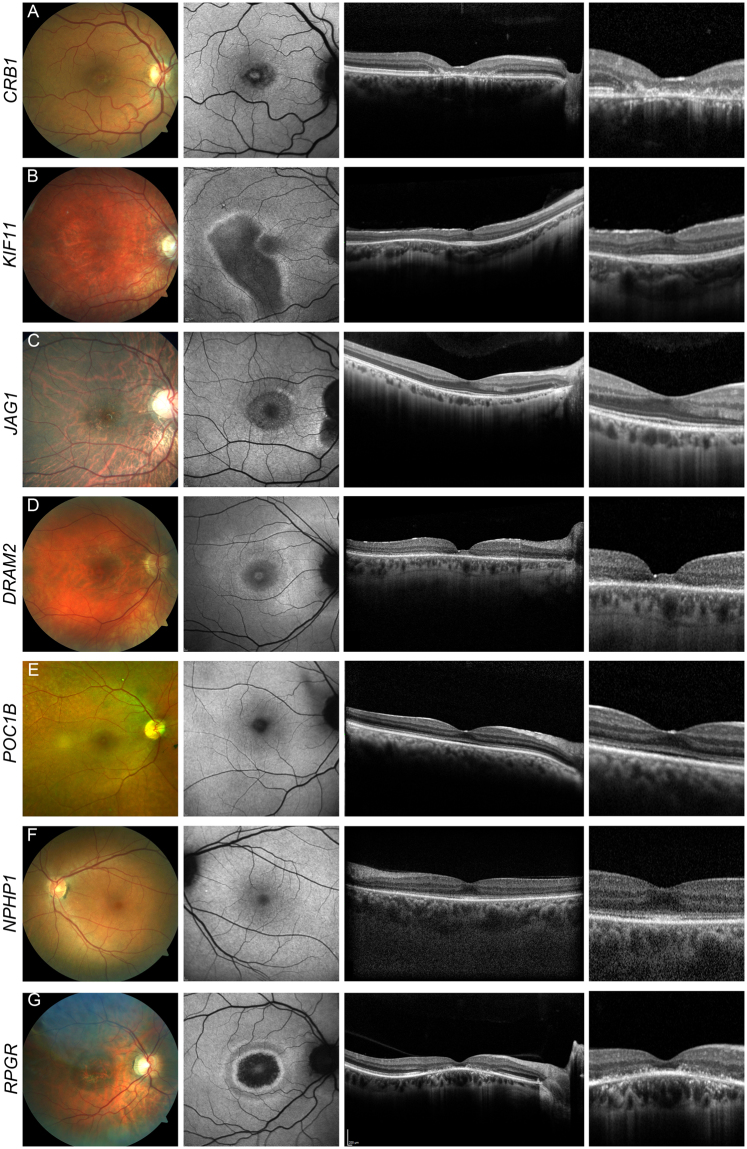
Uncommonly observed but characteristic genotype-phenotype correlations. Retinal phenotype associated with mutations in *CRB1* [#117] (**A**), *KIF11* [#179] (**B**), *JAG1* [#184] (**C**), *DRAM2* [#119] (**D**), *POC1B* [#120] (**E**), *NPHP1* [#115] (F), *RPGR* [#189] (**G**). Fundus color image (first column), fundus AF with 488 nm excitation light (second column), and horizontal spectral-domain OCT (third and fourth column) are shown. Patient numbers refer to Supplementary Table 1. Only one eye is shown due to high symmetry between eyes.

A 57 year-old female patient (#117, Supplementary Table 1, Fig. 2A) with macular dystrophy reported first symptoms (reduced visual acuity) at the age of 49 years. Now, BCVA was 20/50 and 20/400 in the right and left eye, respectively. Retinal imaging revealed a (para-) central loss of photoreceptors and reduction of fundus AF (Fig. 2A). Full-field ERG recordings were within normal limits. We identified a homozygous in frame deletion in *CRB1* previously described in patients with early-onset retinal dystrophy^13,14^. There were no further affected individuals in the family history, and no family members were available for segregation analysis.

#### *KIF11*

Figure 2B shows the retinal phenotype in an apparently non-syndromic 11 year-old patient (#179, Supplementary Table 1) with a heterozygous in frame deletion in the *KIF11* gene. Mutations in this gene are otherwise known to cause an autosomal dominant syndrome including microcephaly, mental retardation, lymphedema and focal chorioretinal atrophy at the ocular fundus^15–17^, and familial exudative vitreoretinopathy^18,19^. Fundus AF imaging revealed areas with a decreased signal surrounded by a line of increased AF and associated with thinning of the photoreceptor layer (Fig. 2B). Similar but more progressed findings were identified in an additional patient (#180, Supplementary Table 1) with a heterozygous nonsense mutation in *KIF11*. A detailed characterization of *KIF11*-associated retinopathy including these patients has recently been reported^20^. The retinopathy was initially classified as non-syndromic due to the very mild systemic manifestations that may be too subtle to be noted in routine ophthalmologic workup.

#### *JAG1*

Figure 2C shows the phenotype of a 24 year-old patient (#184, Supplementary Table 1) with Bull’s eye retinopathy associated with a heterozygous 4-bp deletion in *JAG1*, a gene commonly associated with autosomal dominant Alagille syndrome^21^. The retinal phenotype of Alagille syndrome is variable and includes granular pigmentary changes and maculopathy^22–24^. The patient experienced a mild reduction in visual acuity (BCVA now 20/25 in the right eye and 20/32 in the left eye) and dark adaption problems. In addition, she also had diverse heart defects including a double-outlet-right-ventricle (cardiac surgery at the age of six years), pulmonary stenosis, and a severe scoliosis for which she underwent spinal surgery. These heart defects had not been seen in a syndromic context with her retinal changes before genetic testing, and sequencing of *PTPN11* and *KRAS* for suspected Noonan syndrome had revealed no mutation. In her family, no visual problems or other diseases associated with Alagille syndrome were known. Only the mother was available for segregation analysis and did not carry the *JAG1* mutation. Notably, mutations in *JAG1* frequently occur *de novo*^25–27^.

#### *DRAM2*

Figure 2D illustrates the retinal phenotype of a 53 years-old patient (#119, Supplementary Table 1) with compound heterozygous missense mutations in *DRAM2*. *DRAM2* mutations have been found to cause a retinal dystrophy with early macular involvement^28,29^. The patient experienced dark adaption difficulties starting in her third decade and visual field defects for the past two years. Increased glare and reduced visual acuity were noted for one year. Epiretinal membrane and cataract surgery were performed in her left eye some months ago without functional success. BCVA was now 20/32 and 20/800 in the right and left eye, respectively. Funduscopy revealed a granular macular appearance, and fundus AF showed a paracentral hyperautofluorescent ring with central thinning of the photoreceptor layer. Scotopic and photopic responses on ffERG recordings were reduced to about 2/3 of the lower normal limits. There were no further affected individuals in the family history, and no family members were available for segregation analysis.

#### *POC1B*

Figure 2E shows the retinal phenotype of a non-syndromic 49 years-old patient (#120, Supplementary Table 1) with a homozygous missense mutation in the *POC1B* gene (parents are second degree cousins). Mutations in *POC1B* have been associated with severe and slowly progressive CRD, and with Joubert syndrome with Leber congenital amaurosis (LCA)^30–32^. The patient experienced reduced visual acuity since childhood, and BCVA now was 20/200 in the right eye and 20/400 in the left eye. Increased glare and color vision abnormalities were noted at 18 years of age, but she had no visual difficulties in dim lightening. Fundus AF imaging revealed mild granular irregularities associated with thinning of the photoreceptor layer on OCT imaging. In the mid periphery, there was a faintly noticeable whitish patchy/flecked appearance. Photopic and scotopic responses on full-field ERG testing were undetectable. There were no further affected individuals in the family history, and no family members were available for segregation analysis.

#### *NPHP1*

Figure 2F shows the retinal phenotype of a 49 years-old patient (#115, Supplementary Table 1) with a homozygous deletion of all coding exons of *NPHP1*, a gene commonly associated with non-syndromic nephronophthisis, Senior-Loken or Joubert syndrome^33–35^. Since childhood, the patient experienced reduced visual acuity, glare and, over the last ten years, progressive loss of visual acuity. BCVA now was 20/63 and 20/40 in the right and left eye, respectively. Fundus AF imaging was relatively unremarkable, but OCT imaging revealed diffuse thinning of the outer retinal layers. Scotopic responses on ffERG testing were reduced to about ½ of the lower normal limits and photopic responses were almost extinguished. Only after the genetic test result was discussed with the patient, she disclosed that kidney transplantation had been performed in adolescence due to juvenile-onset nephronophthisis, allowing the diagnosis of a Senior-Loken syndrome. No family members were available for clinical examination or segregation analysis.

#### *RPGR*

Figure 2G shows the retinal phenotype of a patient with sporadic CRD and mutation in *RPGR* (Supplementary Table 1; hemizygous readthrough mutation in patient #189), a gene usually associated with X-linked RP^36–38^ and less commonly with CRD^39–42^. The 41 year-old patient reported reduced visual acuity starting in his 5^th^ decade of life. BCVA was now 20/40 in the right eye and counting fingers due to amblyopia in the left eye. On ffERG testing, scotopic and photopic responses were reduced to about 1/3 of the lower normal limits. No family members were available for segregation analysis or clinical examination.

### Potential novel genotype-phenotype correlations

Potentially disease-causing mutations were detected in six genes which have as yet not been described in association with the observed MD/CCRD phenotype: *FAM161A*, *INPP5E*, *MERTK*, *FBLN5*, *SEMA4A* and *IMPDH1* (n = 1 in all cases). Inclusion of these potential novel genotype-phenotype correlations would increase the diagnostic yield to 76% (n = 191).

#### *FAM161A*

**Figure 3:**
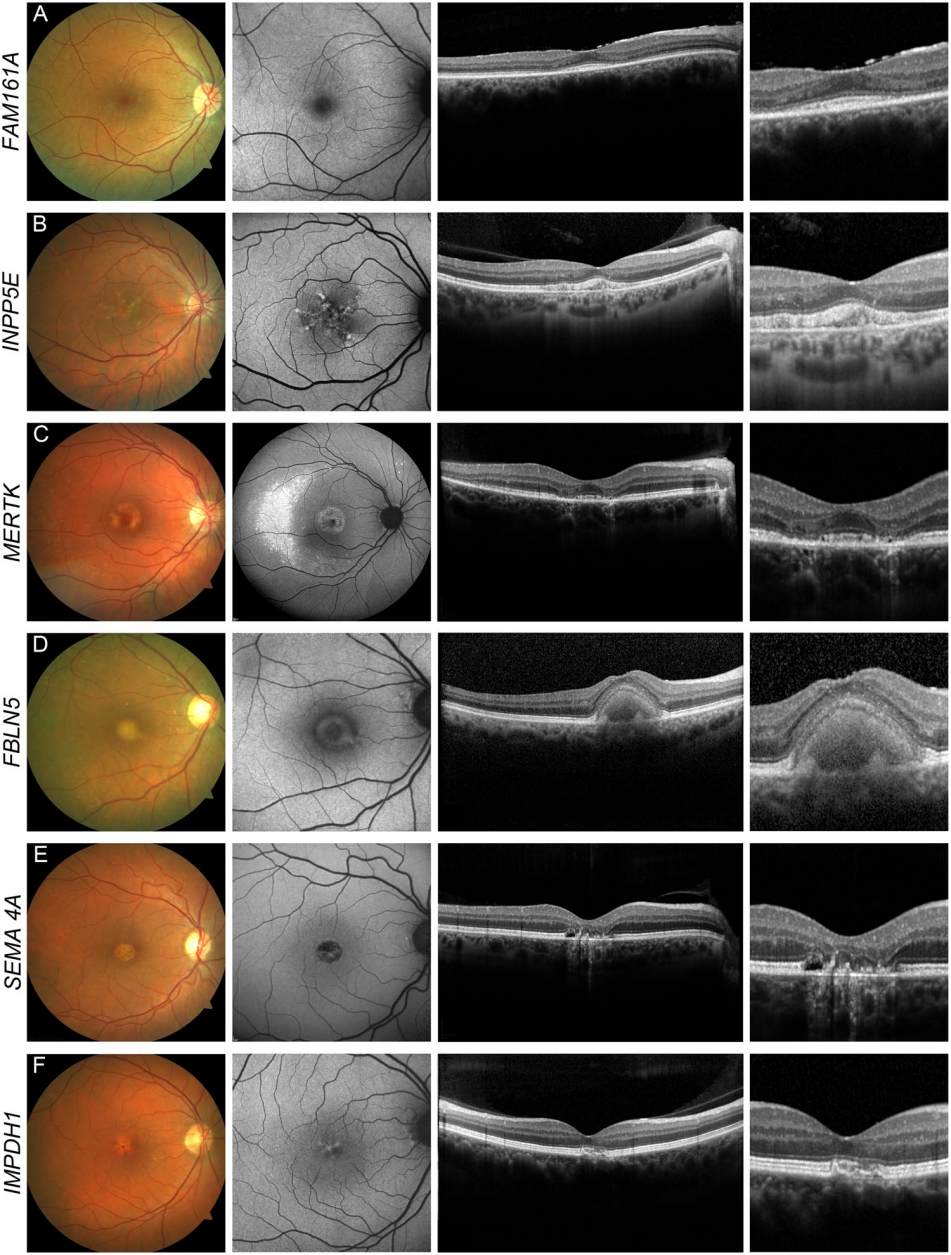
Potential novel genotype-phenotype correlations. Potential novel genotype-phenotype correlations in *FAM161A* [#122] (**A**), *INPP5E* [#121] (**B**), *MERTK* [#113] (**C**), *FBLN5* [#187] (**D**)*, SEMA4A* [#188] (**E**) and *IMPDH1* [#186] **(F**). Fundus color image (first column), fundus AF with 488 nm excitation light (second column), and horizontal spectral-domain OCT (third and fourth column) are shown. Patient numbers refer to Supplementary Table 1. Only one eye is shown due to high symmetry between eyes.

A 54-year-old patient (#122, Supplementary Table 1) reported reduced visual acuity and reading problems around 50 years of age. Two years later, she also noticed dark adaption problems and alterations in color vision. Her visual acuity was 20/32 in both eyes. Scotopic responses on ffERG testing were reduced to about ½ of the lower normal limits and photopic responses reduced to about 1/10 of the lower limit. On OCT imaging, a perifoveal loss of the myoid zone and thinning of the photoreceptor layer was detected, and fundus AF imaging revealed a slightly increased perifoveal autofluorescence (Fig. 3A). NGS identified a novel homozygous 1-basepair deletion in the *FAM161A* gene. The parents of the patient and the only child were deceased. The twelve years older brother had no ocular problems, but was not available for clinical examination or genetic testing.

#### *INPP5E*

A 57 years-old patient (#121, Supplementary Table 1) with a pattern dystrophy reported reduced visual acuity and increased sensitivity to light for the past 3 years. Funduscopy showed macular subretinal yellowish deposits which were associated with a pattern of increased and decreased fundus AF (Fig. 3B). Visual acuity was 20/20 in both eyes, and ERG examination revealed responses within the normal range. NGS identified compound heterozygosity for a nonsense mutation and a missense variant (c.844 G > A; p.Gly282Arg) affecting an evolutionarily highly conserved amino acid in *INPP5E*. The minor allele frequency of the missense variant is low (0.01%), and no homozygotes have been documented in the general population. The patient was otherwise healthy, specifically without signs of Joubert syndrome, which has previously been associated with mutations in *INPP5E*^43–46^. Only the patient’s mother was available for segregation analysis and carried only the nonsense mutation.

#### *MERTK*

A 39 years-old patient (#113, Supplementary Table 1) was affected by increased sensitivity to light and dark adaption problems for about the past 3 years. At initial presentation, BCVA was 20/20 and 20/25 in the right and left eye, respectively, but decreased in the left eye to 20/2000 over a period of two years. Funduscopy showed central hypo- and hyperpigmentations (L > R) and, in the far periphery temporal inferior, scattered bone spicule pigmentations. Fundus AF imaging revealed an uncommon pattern with central granular irregularities and a hyperfluorescent area mainly temporal to the macula with thinning of the photoreceptor layer (Fig. 3C) which progressed over a 5-years observation period. Scotopic and photopic responses on ffERG recordings were reduced to about 2/3 of the lower normal limits. NGS revealed two novel missense variants in *MERTK* (c.1801G > C/p.Val601Leu and c.2360 G > A/p.Gly787Asp) in compound heterozygous state (segregation analysis of the parents), both affecting highly conserved protein residues. Only p.Val601Leu has a (very low) documented minor allele frequency (0.00041%).

#### *FBLN5*

A 78 years-old patient (#187, Supplementary Table 1) reported reduced visual acuity over the past year, and BCVA now was 20/50 in both eyes. Funduscopy showed mild drusen at the vascular arcades and foveal yellowish subretinal deposits with associated increased fundus AF (vitelliform lesion; Fig. 3D). The Arden ratio was 2.0 in both eyes. The patient carried a rare (minor allele frequency of 0.0037%) heterozygous and potentially pathogenic missense variant (c.1093 A > G; p.Ile365Val) in *FBLN5*, affecting an evolutionarily conserved residue of the FBLN5 protein^47^. The patient’s deceased mother and a maternal cousin also had late onset visual problems, but no fundus images and no DNA samples were available for segregation analysis.

#### *SEMA4A*

A novel heterozygous frameshift insertion in the *SEMA4A* gene was identified in a 48-year-old patient (#188, Supplementary Table 1) whose first symptoms were reduced visual acuity noted about four years ago. BCVA was 20/63 and 20/40 in the right and left eye, respectively. Funduscopy and OCT imaging showed an atrophic macular lesion associated with decreased fundus AF (Fig. 3E). On ffERG recording, scotopic responses were reduced to about 2/3 and photopic to about ½ of the lower normal limits. The Arden ratio was 1.8 in both eyes. The patient’s parents were deceased and therefore, segregation analysis was not possible.

#### *IMPDH1*

A 58-year-old patient (#186, Supplementary Table 1) was affected by increasing metamorphopsia for several months and increased glare for decades. BCVA was 20/20 in both eyes and ffERG recordings were within normal limits. Fundus examination, OCT and fundus AF imaging revealed features of a mild pattern dystrophy (Fig. 3F). We identified a novel, heterozygous, synonymous mutation in the *IMPDH1* gene which affects the second-to-last nucleotide of exon 2, predicted to impair the adjacent donor splice site (minor allele frequency 0.05%). Mutations in *IMPDH1* have previously been associated with a.d. retinitis pigmentosa^48–50^. The mother of the patient also had reduced vision beginning in the fourth decade of life. However, the patient’s close relatives were all deceased and therefore, segregation analysis was not possible.

### Phenotype of unsolved patients

Out of the remaining 60 patients, 7 patients (12%) revealed one potentially disease-causing variant in a gene causing recessive retinopathy in combination with a phenotype usually associated with mutations in the respective gene (*ABCA4*, n = 5; *CDH3*, n = 1; C*ERKL*, n = 1; Supplementary Table 4). Seven out of the remaining 53 patients carried at least one previously described *ABCA4* mutation, but showed no phenotype characteristics for *ABCA4*-associated retinopathy.

All 60 genetically unsolved patients were reviewed in order to identify groups with common phenotypic characteristics on fundus photography, OCT- and fundus AF imaging. This allowed to cluster 41 patients into 4 subgroups, each with a phenotypic spectrum resembling 1) mitochondrial retinopathy (n = 10), 2) adult vitelliform or pattern dystrophy (n = 15), 3) late-onset retinal dystrophy (LORD) or Sorsby fundus dystrophy (n = 7) or 4), *PRPH2*- or *ABCA4*-associated retinopathy (n = 9). The remaining 19 patients showed less frequent phenotypes and could not be assigned to any of these subgroups.

All patients with a phenotype typical for mitochondrial retinopathies showed changes compatible with those described for the *MT-TL1* mutation (m.3243 A > G)^51^, such as pigmentary abnormalities, flecks of increased AF with borders of decreased AF and/or chorioretinal atrophy. However, none of these 10 patients carried the m.3243 A > G variant. A muscle biopsy in 3 of the 10 patients revealed characteristics of a mitochondrial disease, and two patients exhibited further clinical characteristics such as deafness, cardiac problems and a family history suggestive for a mitochondrial disease.

## Discussion

Our study demonstrates a high detection rate of disease-causing mutations in MD/CCRD patients using targeted NGS and confirms the extensive genetic heterogeneity of this disease group in 251 MD/CCRD patients. The study’s unique feature is the in-depth clinical characterization of all patients regardless of the genetic testing results, including the “unsolved”. Moreover, the unselected cohort of consecutive patients provides a realistic insight into the distribution of MD/CCRD in a routine clinical setting.

The overall diagnostic yield of 74% achieved in our study is in the upper range compared to previous reports of targeted NGS in MD/CCRD patients^2,9,10^. However, the study by Stone *et al*. had a significantly higher detection rate in the subgroup of patients with macular dystrophies^11^. Reasons for discrepancy between yields may include different cohort sizes and populations, variable inclusion criteria and the investigation of specific subgroups. Moreover, efficiency of molecular genetic testing and data analysis may differ between studies.

### Mutations in genes known to be involved in MD/CCRD pathogenesis

Mutations in *ABCA4*, *PRPH2* and *BEST1* were predominant, explaining the retinal disease in 57% of the entire cohort and 74% of the solved cases. Although referral patterns might have biased our cohort towards a slightly exaggerated frequency of *ABCA4*-associated retinopathy, previous studies have likewise indicated that mutations in *ABCA4* are the most common cause of central retinal dystrophies^2,11,52–54^.

In a large French CCRD cohort, *PRPH2* mutations were found in 3.4% of all cases (in 11.8% of a.d. patients)^2^, compatible with other studies^9,55^. Thus, the high proportion of MD/CCRD patients with *PRPH2* mutations (12% of all cases) in this study is surprising. Many patients with *PRPH2* mutation present with a phenotype similar to late-onset Stargardt disease. Such patients may often be preferentially tested only for *ABCA4* mutations, or be diagnosed with age-related macular degeneration and therefore receive no genetic diagnosis.

Some of the genes whose mutations are associated with isolated or syndromic MD/CCRD have been reported only rarely, and sometimes without in-depth characterization of the retinal phenotype. Targeted NGS allows to include these genes in the molecular analysis with only minor additional resources when compared with Sanger sequencing. Phenotypic re-evaluation after identification of the (likely) molecular basis of the disease revealed in the vast majority of cases that the respective retinal phenotype was in line with previously published and often characteristic genotype-phenotype correlations on multi-modal imaging (Fig. 2). This may not only increase the confidence in a correct molecular diagnosis, but also confirms previous rare phenotypic disease characterizations for genes infrequently involved in the pathophysiology of MD/CCRD.

Such rare genotype-phenotype correlations were confirmed in this report in patients with mutations in *CRB1, DRAM2, KIF11, JAG1, POC1B, NPHP1* and *RPGR*. Mutations in *CRB1* mainly account for LCA and RP^13,56–59^ and have only recently been described in association with isolated MD^60,61^. The herein reported homozygous *CRB1* mutation was previously reported in a patient with EOSRD^13,62^. Occurrence in a patient with late-onset MD (Fig. 2A) may suggest that other (most likely genetic) factors might influence the disease manifestation. The patients with mutations in genes associated with syndromic MD/CCRD all showed a retinal phenotype compatible with the previously described syndrome-associated retinopathy. These patients either revealed no other obvious systemic abnormalities (*POC1B*), showed only very mild syndromic changes that may easily escape attention (*KIF11*)^20^, or received their precise syndromic diagnosis subsequent to the identification of the mutation causing their retinopathy (*JAG1, NPHP1*). Thus, analysis of genes causing syndromic retinal dystrophies in patients who are primarily diagnosed with an isolated retinopathy may clarify the exact clinical diagnosis.

The identification of a patient with a mutation in *RPGR* – mostly causing X-linked RP^36–38^ – confirms its rarer association with MD/CCRD^39–41^. Sporadic *RPGR*-associated CRD is rare^63^, and our case supports the importance to consider *RPGR* mutations in this specific subgroup. Two additional patients in our cohort were found to have either an in-frame deletion (c.2203_2226del) or duplication (c.2919_2939dup) in *ORF15* of *RPGR*, which were not interpreted as disease-causing due to the relatively frequent occurrence of such variants in the normal population, although the patient with the duplication revealed a phenotype compatible with *RPGR*-related retinopathy. Retinal phenotyping of obligate female carriers and segregation analysis would be desirable for confirming pathogenicity of identified mutations in future studies, but this was not possible in our patients.

### Potential novel genotype-phenotype correlations

One of the advantages of targeted NGS is the possibility to screen a large number of genes involved in the pathophysiology of retinal dystrophies independent of the exact phenotype. In 6 (2%) patients, potentially disease-causing variants were detected in genes for which an association with the observed MD/CCRDs phenotype has not previously been established. In these cases, it remains challenging to confirm or exclude the pathogenicity of identified variants in a routine clinical setting. In recessive traits, with nowadays small and highly mobile Western families, only one affected disease carrier is often identified which limits the confidence to establish novel genotype-phenotype correlations, even when parental segregation analysis is performed. In dominant traits, mostly in those with a late-onset of the disease, small families with no further living disease carriers also limit the interpretability of the genetic findings. In absence of functional assessment and/or animal models for these individual genetic variants, future observations may eventually confirm or disprove such potential novel genotype-phenotype correlations. We hope to facilitate such future comparisons by providing essential phenotype information.

We identified patients exhibiting features of a pattern dystrophy (*IMPDH1*), or non-syndromic CRD (*INPP5E* and *FAM161A*). These genes are otherwise associated with a.r. (*FAM161A*^64–69^) or a.d. (*IMPDH1*^48–50^) retinitis pigmentosa, or Joubert syndrome (*INPP5E*^43–45^). Of note, the patient with *FAM161A* mutations had a late onset of first symptoms (reduced visual acuity at the age of 50 years) which was followed 2 years later by dark adaption problems. Although ffERG currently indicated CRD, the phenotype might be classified as RP later in the disease course. There is little published information on the *INPP5E-*related retinal phenotype, but the report by Hardee *et al*. on *INPP5E-*related Joubert syndrome indicates a retinal dystrophy that primarily affects the central retina/cone system^46^. There is a lack of detailed phenotypic description of a.d. *SEMA4A*-associated retinopathy^70,71^, and the as yet reported *MERTK*-associated phenotype^72–74^ differs on retinal imaging and with regards to the age-of-onset from the patient reported herein. Despite the less consistent pathogenicity prediction of the *FBLN5* variant, the phenotype may be in line with previous reports^47^.

### Unsolved cases

There are still a substantial number of patients in which targeted NGS is not effective in confirming the clinical diagnosis on a molecular level. Common phenotypes in a subset of these patients may indicate that specific mutations, genes or pathways are involved. Recognizing such phenotypic patterns in mutation negative patients might be helpful to collaboratively build more substantial cohorts despite the rarity of the individual cases for identifying novel disease causes not detected with the current diagnostic approach.

We identified four phenotypic subgroups similar to 1) mitochondrial retinopathy, 2) adult vitelliform or pattern dystrophy, 3) fundus dystrophy similar to LORD or Sorsby fundus dystrophy or 4) *PRPH2*- or *ABCA4*-related retinopathy. Other phenotypes with less frequent and possibly specific fundus features might represent retinal dystrophies with as yet unknown, rare genetic cause.

## Summary

We demonstrate a high detection rate of mutations in a representative MD/CCRD cohort of 251 consecutive patients and confirm the genetic heterogeneity of MD/CCRD. Targeted NGS is efficient in detecting even unexpected or rare genetic disease causes and might facilitate the identification of novel genotype-phenotype correlations.

## Electronic supplementary material

Supplementary tables
