# Supplementary material for: Clinical and genetic characteristics of 251 consecutive patients with macular and cone/cone-rod dystrophy
Source: Sci Rep. 2018 Mar 19;8:4824. doi: 10.1038/s41598-018-22096-0 (PMC5859282; doi:10.1038/s41598-018-22096-0)
Supplement: Supplementary file 1 — Supplementary tables [file 41598_2018_22096_MOESM1_ESM.pdf]

Clinical and genetic characteristics of 251 consecutive patients with macular and cone/cone-rod dystrophy

Johannes Birtel[1,2], Tobias Eisenberger[3], Martin Gliem[1,2], Philipp L. Müller[1,2], Philipp Herrmann[1,2], Christian Betz[3], Diana Zahnleiter[3], Christine Neuhaus[3], Steffen Lenzner[3], Frank G. Holz[1,2], Elisabeth Mangold[4], Hanno J. Bolz[3,5]*, Peter Charbel Issa[1,2,6]*

[1] Department of Ophthalmology, University of Bonn, Bonn, Germany
[2] Center for Rare Diseases Bonn (ZSEB), University of Bonn, Bonn, Germany
[3] Bioscientia Center for Human Genetics, Ingelheim, Germany
[4] Institute of Human Genetics, University of Bonn, Bonn, Germany
[5] Institute of Human Genetics, University Hospital of Cologne, Cologne, Germany
[6] Oxford Eye Hospital, Oxford University Hospitals NHS Foundation Trust, and Nuffield Laboratory of Ophthalmology, Department of Clinical Neurosciences, University of Oxford, Oxford, UK

Supplementary Table 1

Epidemiological and clinical characteristics including mutations identified in this study.

ID (#)	Gender (m/f)	Age of onset	First symptoms	Age at examination	EOG	ERG scotopic	ERG photopic	Panel	Gene	Inherit-ance	Genotype	Exons/Introns (IVS)	Nucleotide	Protein	Reference
**Autosomal recessive**															
1	w	18	rva	44	np	nor	nor	Sanger	*ABCA4*	sp	het<br>het	Exon 28<br>Exon 42	c.4234C>T<br>c.5882G>A	p.Gln1412*<br>p.Gly1961Glu	[1-3]<br>[4-6]
2	w	50	rva	51	np	nor	nor	NGS	*ABCA4*	sp	het<br>het	Exon 22<br>Exon 42	c.3322C>T<br>c.5882G>A	p.Arg1108Cys<br>p.Gly1961Glu	[2,7,8]<br>[4-6]
3	w	27	rva	34	np	np	np	Sanger	*ABCA4*	ar	het<br>het	Exon 39<br>Exon 42	c.5512C>G<br>c.5882G>A	p.His1838Asp<br>p.Gly1961Glu	[9]<br>[4-6]
4	w	6	rva	11	np	bor	red	Sanger	*ABCA4*	ar	hom	Exon 49	c.6746C>A	p.Ala2249Asp	novel
5	w	60	rva	76	np	np	np	Sanger	*ABCA4*	sp	het<br>het<br>het<br>het	Exon 3<br>Exon 40<br>Intron 33<br>Exon 40	c.1822T>A<br>c.5603A>T<br>c.4773+48C>T<br>c.5682G>C	p.Phe608Ile<br>p.Asn1868Ile<br>non coding, p.c.u.<br>p.Leu1894Leu	[10-12]<br>[12,13]<br>[14]<br>[15] *
6	m	18	rva	20	nor	bor	red	NGS	*ABCA4*	sp	het<br>het<br>het	Exon 10<br>Exon 17<br>Exon 40	c.1253T>C<br>c.2588G>C<br>c.5603A>T	p.Phe418Ser<br>p.Gly863Ala<br>p.Asn1868Ile	[16]<br>[1,5,17]<br>[12,13]
7	m	60	rva	60	np	nor	red	Sanger	*ABCA4*	sp	het<br>het<br>het<br>het<br>het	Exon 1<br>Exon 12<br>Exon 17<br>Exon 19<br>Exon 40	c.52C>T<br>c.1715G>A<br>c.2588G>A<br>c.2828G>A<br>c.5603A>T	p.Arg18Trp<br>p.Arg572Gln<br>p.Gly863Ala<br>p.Arg943Gln<br>p.Asn1868Ile	[9,18]<br>[10]<br>[1,5,17]<br>[13,19]<br>[12,13]
8	w	9	rva	17	np	red	red	NGS	*ABCA4*	ar	het<br>het	Exon 39<br>Exon 39	c.5509C>A<br>c.5549T>C	p.Pro1837Thr<br>p.Leu1850Pro	novel<br>[20]
9	w	23	rva	32	np	red	red	Sanger	*ABCA4*	sp	het<br>het<br>het<br>het	Exon 12<br>Exon 33<br>Exon 12<br>Exon 21	c.1654G>A<br>c.4771G>A<br>c.1622T>C<br>c.3113C>T	p.Val552Ile<br>p.Gly1591Arg<br>p.Leu541Pro<br>p.Ala1038Val	[21]<br>[22]<br>[4,12,13]<br>[4,12,13]
10	w	13	rva	15	np	nor	red	Sanger	*ABCA4*	ar	het<br>het	Exon 10<br>Exon 42	c.1309del<br>c.5882G>A	p.Gln437Argfs*12<br>p.Gly1961Glu	[22]<br>[4-6]
11	w	32	rva	35	np	red	red	NGS	*ABCA4*	sp	het<br>het	Exon 21<br>Exon 28	c.3113C>T<br>c.4234C>T	p.Ala1038Val<br>p.Gln1412*	[4,6,23]<br>[1-3]
12	w	23	rva	29	np	nor	red	Sanger	*ABCA4*	sp	het<br>het	Exon 3<br>Exon 42	c.206G>A<br>c.5882G>A	p.Trp69*<br>p.Gly1961Glu	[24]<br>[4-6]
13	w	35	rva	40	np	nor	red	NGS	*ABCA4*	sp	het<br>het	Exon 42<br>Exon 35	c.5882G>A<br>c.4873C>T	p.Gly1961Glu<br>p.His1625Tyr	[4-6]<br>[25]
14	w	20	rva	40	np	nor	nor	NGS	*ABCA4*	sp	het<br>het	Exon 6<br>Exon 42	c.768G>T<br>c.5882G>A	splice site<br>p.Gly1961Glu	[1,26]<br>[4-6]
15	w	39	rva	67	np	red	red	NGS	*ABCA4*	sp	het<br>het<br>het<br>het	Exon 13<br>Exon 6<br>Exon 17<br>Exon 40	c.1830T>A<br>c.656G>C<br>c.2588G>C<br>c.5603A>T	p.Tyr610*<br>p.Arg219Thr<br>p.Gly863Ala<br>p.Asn1868Ile	novel<br>[27]<br>[1,5,17]<br>[12,13]
16	w	35	rva	45	np	np	np	Sanger	*ABCA4*	ar	het<br>het	Exon 16<br>Exon 28	c.2401G>A<br>c.4234C>T	p.Ala801Thr<br>p.Gln1412*	[28]<br>[1-3]
17	m	chi	rva	28	np	red	ext	Sanger	*ABCA4*	sp	hom	Exon 30	c.4462T>C	p.Cys1488Arg	[4,10,12]
18	w	14	rva	18	np	nor	red	Sanger	*ABCA4*	sp	het<br>het	Exon 1<br>Exon 42	c.45G>A<br>c.5882G>A	p.Trp15*<br>p.Gly1961Glu	[1,3]<br>[4-6]

19	m	43	rva	54	np	np	np	Sanger	*ABCA4*	sp	het<br>het	Exon 35<br>Exon 40	c.4918C>T<br>c.5693G>A	p.Arg1640Trp<br>p.Arg1898His	[29]<br>[17]
20	w	27	rva	29	np	nor	nor	Sanger	*ABCA4*	sp	het<br>het	Exon 42<br>Exon 43	c.5882G>A<br>c.5917del	p.Gly1961Glu<br>p.Val1973*	[4-6]<br>[4]
21	m	7	rva	35	np	red	ext	NGS	*ABCA4*	ar	hom	Exon 30	c.4462T>C	p.Cys1488Arg	[4,10,12]
22	w	19	rva	66	nor	nor	nor	Sanger	*ABCA4*	ar	het<br>het<br>het	Exon 42<br>Exon 29<br>Exon 46	c.5882G>A<br>c.4297G>A<br>c.6326T>C	p.Gly1961Glu<br>p.Val1433Ile<br>p.Leu2109Pro	[4-6]<br>[10]<br>[30]
23	m	44	rva	49	np	bor	red	Sanger	*ABCA4*	sp	het<br>het	Exon 22<br>Exon 40	c.3272G>A<br>c.5603A>T	p.Gly1091Glu<br>p.Asn1868Ile	[7]<br>[12,13]
24	w	18	rva	37	np	red	red	Sanger	*ABCA4*	sp	het<br>het	Exon 28<br>Exon 43	c.4195G>A<br>c.5929G>A	p.Glu1399Lys<br>p.Gly1977Ser	[4]<br>[4]
25	w	30	rva	56	np	red	red	NGS	*ABCA4*	ar	het<br>het<br>het	Exon 12<br>Exon 21<br>Exon 13	c.1622T>C<br>c.3113C>T<br>whole-exon deletion	p.Leu541Pro<br>p.Ala1038Val<br>putative loss of function	[4,12,13]<br>[4,12,13]<br>[24]
26	w	32	rva	42	np	nor	red	NGS	*ABCA4*	sp	het<br>het	Exon 16<br>Exon 1	c.2401G>A<br>c.45G>A	p.Ala801Thr<br>p.Trp15*	[28]<br>[1]
27	w	19	rva	39	np	np	np	Sanger	*ABCA4*	sp	het<br>het<br>het<br>het	Exon 17<br>Exon 40<br>Exon 12<br>Exon 21	c.2588G>C<br>c.5603A>T<br>c.1622T>C<br>c.3113C>T	p.Gly863Ala<br>p.Asn1868Ile<br>p.Leu541Pro<br>p.Ala1038Val	[1,5,17]<br>[12,13]<br>[4,12,13]<br>[4,12,13]
28	w	31	rva	36	np	nor	red	Sanger	*ABCA4*	ar	het<br>hom	Exon 39<br>Exon 42	c.5512C>G<br>c.5882G>A	p.His1838Asp<br>p.Gly1961Glu	[9]<br>[4-6]
29	w	11	rva	14	np	bor	red	NGS	*ABCA4*	sp	het<br>het	Exon 3<br>Exon 17	c.194G>A<br>c.2588G>C	p.Gly65Glu<br>p.Gly863Ala	[31]<br>[1,5,17]
30	w	44	rva	55	np	nor	red	Sanger	*ABCA4*	sp	het<br>het	Exon 11<br>Exon 13	c.1411G>C<br>c.1903C>T	p.Glu471Lys<br>p.Gln635*	[32]<br>[4]
31	w	24	rva	35	np	na	na	NGS	*ABCA4*	sp	het<br>het	Exon 42<br>Exon 15	c.5882G>A<br>c.2300T>A	p.Gly1961Glu<br>p.Val767Asp	[4-6]<br>[33,34]
32	w	8	rva	16	np	red	red	Sanger	*ABCA4*	sp	het<br>het	Exon 28<br>Intron 38	c.4234C>T<br>c.5461-10T>C	p.Gln412Tyr<br>splice site	[1]<br>[35]
33	w	6	rva	24	bor	nor	bor	Sanger	*ABCA4*	sp	het<br>het	Intron 40<br>Exon 43	c.5714+5G>A<br>c.5917del	splice site<br>p.Val1973*	[36,37]<br>[38]
34	m	46	rva	49	np	nor	red	NGS	*ABCA4*	sp	het<br>het	Exon 30<br>Exon 40	c.4537dup<br>c.5603A>T	p.Gln1513Profs*42<br>p.Asn1868Ile	[38]<br>[12,13]
35	w	31	rva	34	np	bor	red	Sanger	*ABCA4*	sp	het<br>het<br>het	Exon 28<br>Exon 43<br>Exon 44	c.4195G>A<br>c.5929G>A<br>c.6079C>T	p.Glu1399Lys<br>p.Gly1977Ser<br>p.Leu2027Phe	[4]<br>[38]<br>[39]
36	w	50	rva	60	np	np	np	Sanger	*ABCA4*	sp	het<br>hom	Exon 36<br>Exon 40	c.5189G>A<br>c.5603A>T	p.Trp1730*<br>p.Asn1868Ile	novel<br>[12,13]
37	w	44	rva	50	np	np	np	Sanger	*ABCA4*	sp	het<br>het	Exon 29<br>Exons 12-13	c.4347G>T<br>deletion of Exons 12-13	p.Trp1449Cys<br>putative loss of function	[30]<br>novel
38	m	14	rva	39	np	na	na	Sanger	*ABCA4*	sp	het<br>het	Exon 42<br>Exon 12	c.5882G>A<br>c.1584C>A	p.Gly1961Glu<br>p.Tyr528*	[4-6]<br>novel
39	w	12	rva	40	np	red	red	Sanger	*ABCA4*	ar	hom	Exon 36	c.5172G>A	p.Trp1724*	novel
40	m	17	rva	18	np	bor	red	Sanger	*ABCA4*	sp	het<br>het<br>het<br>het	Exon 12<br>Exon 21<br>Exon 17<br>Exon 40	c.1622T>C<br>c.3113C>T<br>c.2588G>C<br>c.5603A>T	p.Leu541Pro<br>p.Ala1038Val<br>p.Gly863Ala<br>p.Asn1868Ile	[4,12,13]<br>[4,12,13]<br>[1,5,17]<br>[12,13]
41	w	38	pho	40	np	nor	red	Sanger	*ABCA4*	sp	het<br>het<br>het	Exon 40<br>Intron 8<br>Exon 8	c.5603A>T<br>c.1937+1G>A<br>c.1009T>C	p.Asn1868Ile<br>splice site<br>p.Phe337Leu	[12,13]<br>[4]<br>[24]
42	w	31	met	62	np	np	np	Sanger	*ABCA4*	ar	het<br>het	Exon 23<br>Exon 36	c.3468C>G<br>c.5059A>T	p.Tyr1156*<br>p.Ile1687Phe	novel<br>[40]
43	m	9	rva	16	np	red	red	Sanger	*ABCA4*	sp	het<br>het	Exon 21<br>Exon 22	c.3098del<br>c.3243G>T	p.Lys1033Serfs*51<br>p.Lys1081Asn	novel<br>novel

44	m	7	rva	17	np	nor	nor	Sanger	*ABCA4*	ar	het<br>het<br>het	Exon 22<br>Exon 12<br>Exon 21	c.3212C>T<br>c.1622T>C<br>c.3113C>T	p.Ser1071Leu<br>p.Leu541Pro<br>p.Ala1038Val	[10]<br>[4,12,13]<br>[4,12,13]
45	w	20	rva	54	np	nor	nor	Sanger	*ABCA4*	ar	het<br>het	Exon 42<br>Intron 36	c.5882G>A<br>c.5196+2T>C	p.Gly1961Glu<br>splice site	[4-6]<br>[10,39]
46	w	20	rva	23	np	red	red	NGS	*ABCA4*	sp	het<br>het	Exon 6<br>Exon 23	c.768G>T<br>c.3386G>T	p.Val256Val<br>p.Arg1129Leu	[26,32]<br>[9,32,41]
47	m	45	rva	47	np	red	red	Sanger	*ABCA4*	sp	het<br>het<br>het	Exon 44<br>Exon 12<br>Exon 21	c.6089G>A<br>c.1622T>C<br>c.3113C>T	p.Arg2030Gln<br>p.Leu541Pro<br>p.Ala1038Val	[38]<br>[4,12,13]<br>[4,12,13]
48	m	24	rva	25	np	nor	nor	NGS	*ABCA4*	sp	het<br>het	Exon 40<br>Exon 47	c.5603A>T<br>c.6419T>G	p.Asn1868Ile<br>p.Leu2140Arg	[12,13]<br>[22]
49	m	45	rva	58	np	nor	nor	Sanger	*ABCA4*	ar	het<br>het	Exon 27<br>Exon 42	c.3871C>T<br>c.5882G>A	p.Gln1291*<br>p.Gly1961Glu	[22,30]<br>[4-6]
50	w	8	rva	10	np	np	np	Sanger	*ABCA4*	sp	het<br>het<br>het	Exon 12<br>Exon 21<br>Exon 13	c.1622T>C<br>c.3113C>T<br>whole-exon deletion	p.Leu541Pro<br>p.Ala1038Val<br>putative loss of function	[4,12,13]<br>[4,12,13]<br>[24]
51	w	13	rva	37	np	nor	nor	Sanger	*ABCA4*	sp	het<br>het	Exons 20-22<br>Exon 17	deletion of Exons 20-22<br>c.2588G>C	putative loss of function<br>p.Gly863Ala	[1]<br>[1,5,17]
52	w	25	rva	29	np	nor	nor	NGS	*ABCA4*	sp	het<br>het	Exon 13<br>Exon 40	c.1822T>A<br>c.5603A>T	p.Phe608Ile<br>p.Asn1868Ile	[10-12]<br>[10-12]
53	m	17	rva	41	np	bor	bor	Sanger	*ABCA4*	sp	het<br>het<br>het	Exon 20<br>Exon 40<br>Intron 40	c.2948C>T<br>c.5603A>T<br>c.5714+5G>A	p.Thr983Ile<br>p.Asn1868Ile<br>splice site	[27]<br>[12,13]<br>[36,37]
54	w	8	rva	23	np	ext	ext	NGS	*ABCA4*	pd	het<br>het<br>het	Exon 12<br>Exon 21<br>Exon 13	c.1622T>C<br>c.3113C>T<br>c.1891G>A	p.Leu541Pro<br>p.Ala1038Val<br>p.Gly631Arg	[4,12,13]<br>[4,12,13]<br>[30]
55	w	24	rva	48	np	nor	red	Sanger	*ABCA4*	sp	het<br>het	Exon 44<br>Exon 48	c.6089G>A<br>c.6545_6580del	p.Arg2030Gln<br>p.Leu2184_Phe2193del	[38]<br>[22]
56	w	10	rva	26	np	na	na	Sanger	*ABCA4*	sp	het<br>het	Exon 13<br>Exon 30	c.1807T>C<br>c.4462T>C	p.Tyr603His<br>p.Cys1488Arg	novel<br>[4,10,12]
57	m	12	rva	31	np	nor	red	Sanger	*ABCA4*	ar	het<br>het	Intron 40<br>Exon 22	c.5714+5G>A<br>c.3261A>C	splice mutation<br>p.Glu1087Asp	[36,37]<br>[23,27]
58	w	25	rva	50	np	bor	red	Sanger	*ABCA4*	sp	het<br>het	Exon 6<br>Exon 40	c.634C>T<br>c.5603A>T	p.Arg212Cys<br>p.Asn1868Ile	[11,18,42]<br>[12,13]
59	w	13	rva	16	np	nor	red	Sanger	*ABCA4*	sp	het<br>het	Exon 42<br>Exon 43	c.5882G>A<br>c.5917del	p.Gly1961Glu<br>p.Val1973*	[4-6]<br>[12]
60	w	25	rva	50	np	red	red	NGS	*ABCA4*	ar	het<br>het<br>hom	Intron 38<br>Exon 17<br>Exon 40	c.5461-10T>C<br>c.2588G>C<br>c.5603A>T	splice<br>p.Gly863Ala<br>p.Asn1868Ile	[23,42]<br>[1,5,17]<br>[12,13]
61	w	17	rva	27	np	nor	red	Sanger	*ABCA4*	sp	het<br>het<br>het	Exon 40<br>Exon 8<br>Exon 46	c.5603A>T<br>c.872C>T<br>c.6310dup	p.Asn1868Ile<br>p.Pro291Leu<br>p.Gln2104Profs*31	[43]<br>[43-45]<br>novel
62	w	25	rva	30	np	nor	red	Sanger	*ABCA4*	sp	het<br>het	Exon 44<br>Exon 11	c.6112C>T<br>c.1523G>C	p.Arg2038Trp<br>p.Arg508Pro	[39,46]<br>novel
63	m	31	haz, met	45	np	bor	bor	Sanger	*ABCA4*	pd	het<br>hom	Exon 22<br>Exon 40	c.3210_3211dup<br>c.5603A>T	p.Ser1071Cysfs*14<br>p.Asn1868Ile	[39]<br>[12,13]
64	m	7	rva	18	np	np	np	Sanger	*ABCA4*	ar	het<br>het<br>het	Exon 28<br>Exon 12<br>Exon 21	c.4234C>T<br>c.1622T>C<br>c.3113C>T	p.Glu1412*<br>p.Leu541Pro<br>p.Ala1038Val	[1]<br>[4,12,13]<br>[4,12,13]
65	m	32	rva	51	np	red	red	NGS	*ABCA4*	sp	het<br>het	Exon 6<br>Exon 40	c.741_744del<br>c.5603A>T	p.Asn247Lysfs*14<br>p.Asn1868Ile	novel<br>[12,13]
66	m	55	rva	61	np	np	np	Sanger	*ABCA4*	sp	het<br>het<br>het	Exon 13<br>Exon 33<br>Exon 28	c.1792G>A<br>c.4771G>A<br>c.4234C>T	p.Val598Met<br>p.Gly1591Arg<br>p.Gln1412*	[22]<br>[22]<br>[1-3]
67	m	12	rva	32	np	nor	nor	Sanger	*ABCA4*	sp	het<br>het<br>het	Intron 40<br>Exon 30<br>Exon 45	c.5714+5G>A<br>c.4463G>A<br>c.6148G>C	splice mutation<br>p.Cys1488Tyr<br>p.Val2050Leu	[36,37]<br>[4]<br>[17,47,48]

68	m	37	rva	45	np	nor	bor	Sanger	*ABCA4*	sp	het<br>het<br>het	Exon 40<br>Exon 45<br>Exon 5	c.5603A>T<br>c.6148G>C<br>c.454C>T	p. Asn1868Ile<br>p.Val2050Leu<br>p.Arg152*	[12,13]<br>[17,47,48]<br>[23,49,50]
69	m	11	rva	13	np	bor	bor	Sanger	*ABCA4*	sp	het<br>het	Exon 14<br>Exon 38	c.2041C>T<br>c.5381C>A	p.Arg681*<br>p.Ala1794Asp	[1,23]<br>[1]
70	w	63	rva	65	nor	nor	nor	NGS	*ABCA4*	sp	het<br>het	Exon 40<br>Exon 45	c.5603A>T<br>c.6229C>T	p.Asn1868Ile<br>p.Arg2077Trp	[12,13]<br>[2,17]
71	w	52	met, pho	55	np	nor	red	NGS	*ABCA4*<br>*ABCA4*<br>*GUCY2D* **	sp	het<br>hom<br>het	Intron 38<br>Exon 40<br>Exon 7	c.5461-10T>C<br>c.5603A>T<br>c.1618C>T	splice site<br>p.Asn1868Ile<br>p.Arg540Cys	[23,42]<br>[12,13]<br>[51]
72	w	35	inf	35	np	nor	nor	Sanger	*ABCA4*	sp	het<br>het<br>het	Exon 40<br>Exon 12<br>Exon 21	c.5603A>T<br>c.1622T>C<br>c.3113C>T	p.Asn1868Ile<br>p.Leu541Pro<br>p.Ala1038Val	[12,13]<br>[4,12,13]<br>[4,12,13]
73	m	8	rva	63	np	ext	ext	NGS	*ABCA4*	sp	het<br>het<br>het	Exon 6<br>Exon 9<br>Exon 40	c.768G>T<br>c.1268A>G<br>c.5603A>T	splice site<br>p.His423Arg<br>p.Asn1868Ile	[1,26]<br>[12,13]<br>[12,13]
74	m	14	rva	22	np	nor	red	Sanger	*ABCA4*	sp	het<br>het	Exon 42<br>Intron 48	c.5882G>A<br>c.6729+19del13	p.Gly1961Glu<br>splice site	[4-6]<br>[3,11,52]
75	m	37	rva	38	np	bor	red	Sanger	*ABCA4*	sp	het<br>hom	Exon 5<br>Exon 40	c.470T>A<br>c.5603A>T	p.Leu157*<br>p.Asn1868Ile	novel<br>[12,13]
76	m	13	rva	15	np	nor	nor	Sanger	*ABCA4*	sp	het<br>het<br>het<br>het	Exon 16<br>Exon 42<br>Intron 38<br>Exon 40	c.2549A>G<br>c.5882G>A<br>c.5461-10T>C<br>c.5603A>T	p.Tyr850Cys<br>p.Gly1961Glu<br>splice site<br>p.Asn1868Ile	[53]<br>[4-6]<br>[23,42]<br>[12,13]
77	w	40	rva	42	np	red	red	Sanger	*ABCA4*	sp	het<br>het<br>het	Exon 30<br>Exon 33<br>Exon 30	c.4468T>C<br>c.4685T>C<br>c.4458delA	p.Cys1490Arg<br>p.Ile1562Thr<br>p.Ser1487Profs*39	novel<br>[14,32,54]<br>Novel
78	w	58	rva	74	np	np	np	Sanger	*ABCA4*	sp	het<br>het	Exon 35<br>Exon 40	c.4854G>T<br>c.5603A>T	p.Trp1618Cys<br>p.Asn1868Ile ‡	novel<br>[12,13]
79	w	8	rva	28	np	red	bor	Sanger	*ABCA4*	sp	het<br>het<br>het	Exon 8<br>Exon 11<br>Exon 23	c.872C>T<br>c.1531C>T<br>c.3482G>A	p.Pro291Leu<br>p.Arg511Cys<br>p.Arg1161His	[43-45]<br>[16,43,44,55]<br>[16,43,44,55]
80	m	5	rva	18	np	red	red	Sanger	*ABCA4*	sp	het<br>het<br>hom	Exon 17<br>Intron 38<br>Exon 40	c.2588G>C<br>c.5461-10T>C<br>c.5603A>T	p.Gly863Ala<br>splice site<br>p.Asn1868Ile	[1,5,17]<br>[23,42]<br>[12,13]
81	m	8	rva	14	np	nor	red	NGS	*ABCA4*	sp	het<br>het<br>het	Exon 17<br>Exon 42<br>Exon 6	c.2626C>T<br>c.5882G>A<br>c.694C>T	p.Gln876*<br>p.Gly1961Glu<br>p.Leu232Phe	[56]<br>[4-6]<br>novel
82	m	5	rva	35	np	np	np	Sanger	*ABCA4*	sp	het<br>het<br>het	Exon 17<br>Exon 40<br>Exon 21	c.2588G>C<br>c.5603A>T<br>c.3085C>T	p.Gly863Ala<br>p.Asn1868Ile<br>p.Gln1029*	[1,5,17]<br>[12,13]<br>[11,57]
83	w	21	rva	62	np	red	red	Sanger	*ABCA4*	pd	het<br>het<br>het	Exon 6<br>Exon 17<br>Exon 40	c.768G>T<br>c.2588G>C<br>c.5603A>T	splice site<br>p.Gly863Ala<br>p.Asn1868Ile	[1,26]<br>[1,5,17]<br>[12,13]
84	w	20	rva	37	np	bor	bor	Sanger	*ABCA4*	ar	het<br>het	Intron 36<br>Exon 42	c.5196+2T>C<br>c.5882G>A	splice site<br>p.Gly1961Glu	[10,39]<br>[4-6]
85	w	53	rva	56	np	nor	nor	Sanger	*ABCA4*	sp	het<br>het	Exon 3<br>Exon 40	c.214G>A<br>c.5603A>T	p.Gly72Arg<br>p.Asn1868Ile	[58]<br>[12,13]
86	m	8	rva	11	np	na	na	Sanger	*ABCA4*	sp	het<br>het	Exon 15<br>Exon 40	c.2255G>A<br>c.5645T>C	p.Ser752Asn<br>p.Met1882Thr	novel<br>[42]
87	w	37	rva, nvp, pho	62	np	np	np	Sanger	*ABCA4*	ar	het<br>het	Exon 27<br>Exon 42	c.3871C>T<br>c.5882G>A	p.Gln1291*<br>p.Gly1961Glu	[22]<br>[4-6]
88	m	54	rva	55	np	np	np	Sanger	*ABCA4*	sp	het<br>het	Exon 8<br>Exon 13	c.872C>T<br>c.1928T>G	p.Pro291Leu<br>p.Val643Gly	[43]<br>[45]
89	w	59	rva	61	np	nor	bor	Sanger	*ABCA4*	ar	het<br>het<br>het	Exon 45<br>Exon 40<br>Intron 36	c.6215G>A<br>c.5603A>T<br>c.5196+1137G>A	p.Ser2072Asn<br>p.Asn1868Ile ‡<br>splice site	[59]<br>[12,13]<br>[60]

90	w	64	rva	66	np	nor	red	Sanger	*ABCA4*	ar	het<br>het	Exon 45<br>Exon 40	c.6215G>A<br>c.5603A>T	p.Ser2072Asn<br>p.Asn1868Ile ‡	[59]<br>[12,13]
91	m	16	rva	36	np	nor	nor	Sanger	*ABCA4*	sp	het<br>het<br>het	Exon 12<br>Exon 21<br>Exon 42	c.1622T>C<br>c.3113C>T<br>c.5882G>A	p.Leu541Pro<br>p.Ala1038Val<br>p.Gly1961Glu	[4,12,13]<br>[4,12,13]<br>[4-6]
92	w	42	rva	55	np	red	red	NGS	*ABCA4*	sp	het<br>het<br>het	Exon 12<br>Exon 21<br>Exon 46	c.1622T>C<br>c.3113C>T<br>c.6320G>A	p.Leu541Pro<br>p.Ala1038Val<br>p.Arg2107His	[4,12,13]<br>[4,12,13]<br>[7,11,44]
93	m	10	rva	11	np	red	red	NGS	*ABCA4*	sp	het<br>het	Intron 33<br>Exon 44	c.4773+3A>G<br>c.6118C>T	splice site<br>p.Arg2040*	[17,60,61]<br>[62]
94	m	23	rva, haz	28	np	red	red	Sanger	*ABCA4*	sp	het<br>het<br>het<br>het	Exon 12<br>Exon 21<br>Exon 17<br>Exon 40	c.1622T>C<br>c.3113C>T<br>c.2588G>C<br>c.5603A>T	p.Leu541Pro<br>p.Ala1038Val<br>p.Gly863Ala<br>p.Asn1868Ile	[4,12,13]<br>[4,12,13]<br>[1,5,17]<br>[12,13]
95	m	50	rva	52	np	red	red	NGS	*CDHR1*	sp	het<br>het	Exon 8<br>Exon 17	c.783G>A<br>c.2522_2528del	p.Pro261<br>p.Ile841Serfs*119	[63]<br>[63]
96	m	55	rva	74	np	na	na	NGS	*CDHR1*	sp	hom	Exon 8	c.783G>A	p.Pro261Pro	[63]
97	m	45	met	64	np	red	red	NGS	*CDHR1*	ar	hom	Exon 8	c.783G>A	p.Pro261Pro	[63]
98	m	46	met	46	np	nor	red	NGS	*CDHR1*	sp	hom	Exon 8	c.783G>A	p.Pro261Pro	[63]
99	m	45	met	50	np	red	red	NGS	*CDHR1*	ar	hom	Exon 8	c.783G>A	p.Pro261Pro	[63]
100	m	43	rva	47	red	red	red	NGS	*CDHR1*	sp	hom	Exon 14	c.1503_1507del	p.Gly502Leufs*32	novel
101	w	36	rva	40	red	nor	bor	Sanger	*BEST1*	sp	hom	Exon 8	c.934G>A	p.Asp312Asn	[64-66]
102	m	18	rva	34	red	red	red	Sanger	*BEST1*	ar	hom	Exon 4	c.422G>A	p.Arg141His	[67]
103	m	10	rva	24	np	red	red	Sanger	*BEST1*	sp	hom	Exon 9	c.956T>C	p.Leu319Pro	novel
104	m	54	rva	62	red	nor	nor	Sanger	*BEST1*	sp	het<br>het	Exon 4<br>Exon 8	c.422G>A<br>c.934G>A	p.Arg141His<br>p.Asp312Asn	[64-66,68]
105	m	30	rva	68	np	nor	red	Sanger	*BEST1*	ar	het<br>het	Exon 5<br>Intron 5	c.584C>T<br>c.636+1G>A	p.Ala195Val<br>splice site	[66,69-71]<br>[66,69-71]
106	w	16	rva	32	np	ext	ext	NGS	*PROM1*	sp	het<br>het	Exon 4<br>Exon 12	c.436C>T<br>c.1354dup	p.Arg146*<br>p.Tyr452Leufs*13	novel<br>novel
107	w	chi	rva	28	np	red	ext	NGS	*PROM1*	ar	hom	Exon 1	c.199C>T	p.Gln67*	novel
108	w	6	rva	44	np	ext	ext	NGS	*PROM1*	sp	hom	Exon 13	c.1354dup	p.Tyr452Leufs*13	novel
109	w	chi	nvp	28	np	na	na	NGS	*PROM1*	ar	hom	Exon 16	c.1853T>G	p.Leu618Arg	novel
110	w	18	rva	24	np	red	red	NGS	*PROM1*	ar	hom	Intron 10	c.1142-1G>A	splice site	[52]
111	m	20	rva	25	np	red	red	NGS	*CERKL*	sp	het<br>het	Exon 6<br>Exon 14	c.847C>T<br>c.1651A>T	p.Arg283*<br>p.Ser551Cys	[72]<br>novel
112	m	21	rva	38	np	np	np	NGS	*CERKL*	sp	het<br>het	Exon 1<br>Exon 2	c.197_200dup<br>whole-exon deletion	p.Leu68Serfs*15<br>putative loss of function	novel<br>novel
113	w	36	pho	39	np	red	red	NGS	*MERTK*	sp	het<br>het	Exon 13<br>Exon 18	c.1801G>C<br>c.2360G>A	p.Val601Leu<br>p.Gly787Asp	novel<br>novel
114	w	chi	rva	17	np	ext	ext	NGS	*MERTK*	ar	het<br>hemi	Exons 2-19<br>Exon 2	deletion of Exons 2-19<br>c.369C>G	putative loss of function<br>p.Tyr123*	novel<br>novel
115	w	chi	rva	49	np	red	red	NGS	*NPHP1*	sp	hom	Deletion	whole gene deletion	loss of function	[73,74]
116	m	17	rva	18	np	nor	bor	NGS	*CDH3*	sp	hom	Exon 11	c.1508G>A	p.Arg503His	[75]
117	w	49	rva	57	np	nor	nor	NGS	*CRB1*	sp	hom	Exon 2	c.498_506del	p.Ile167_Gly169del	[47,76]
118	w	chi	nvp	57	np	red	red	NGS	*RDH5*	ar	hom	Exon 3	c.469C>T	p.Arg157Trp	[77,78]
119	w	27	nvp	53	np	red	red	NGS	*DRAM2*	sp	het<br>het	Exon 3<br>Exon 5	c.47T>C<br>c.284G>T	p.Val16Ala<br>p.Gly95Val	novel<br>novel
120	w	chi	rva	49	np	ext	ext	NGS	*POC1B*	ar	hom	Exon 4	c.317G>C	p.Arg106Pro	[79,80]
121	w	54	rva	57	np	bor	bor	NGS	*INPP5E*	sp	het<br>het	Exon 2<br>Exon 8	c.844G>A<br>c.1629C>A	p.Gly282Arg<br>p.Tyr543*	novel<br>[81]
122	w	50	rva	54	bor	red	red	NGS	*FAM161A*	sp	hom	Exon 3	c.971del	p.Pro324Hisfs*5	novel
**Autosomal dominant**															
123	m	48	rva	56	red	red	red	NGS	*PRPH2*	sp	het	Exon 3	c.920del	p.Leu307Argfs*17	[82,83]
124	w	40	rva	53	red	nor	bor	NGS	*PRPH2*	ad	het	Exon 1	c.2T>C	p.Met1?	[84]
125	w	46	rva	53	np	nor	nor	Sanger	*PRPH2*	ad	het	Exon 2	c.623G>A	p.Gly208Asp	[85-87]

126	w	47	rva	48	np	red	red	Sanger	*PRPH2*	sp	het	Exon 2	c.658C>T	p.Arg220Trp	[88]
127	w	66	rva	76	np	red	red	NGS	*PRPH2*	ad	het	Exon 1	c.571G>T	p.Glu191*	novel
128	w	46	rva	46	np	bor	red	Sanger	*PRPH2*	sp	het	Exon 1	c.515G>A	p.Arg172Gln	[42,89,90]
129	m	54	rva	60	np	red	red	NGS	*PRPH2*	ad	het	Exon 2	c.612C>G	p.Tyr204*	novel
130	w	61	rva	63	np	nor	red	NGS	*PRPH2*	ad	het	Exon 1	c.136C>T	p.Arg46*	[91,92]
131	m	46	rva	51	bor	red	red	NGS	*PRPH2*	ad	het	Exon 1	c.281G>A	p.Trp94*	[93]
132	m	48	rva	56	np	red	red	NGS	*PRPH2*<br>*(RIMS1)*	ad	het<br>het	Exon 2	c.626T>A<br>(c.4945T>A)	p.Val209Asp<br>(p.Ser1649Thr)	novel<br>novel – unclear, if contributing to phenotype
133	w	40	rva	72	np	ext	ext	Sanger	*PRPH2*	ad	het	Exon 1	c.715C>T	p.Gln239*	[85]
134	w	60	met	65	np	red	red	Sanger	*PRPH2*	ad	het	Exon 2	c.771C>G	p.Tyr257*	novel
135	m	15	inf	15	np	bor	red	Sanger	*PRPH2*	sp	het	Exon 1	c.424C>T	p.Arg142Trp	[94]
136	w	63	rva	63	np	bor	bor	Sanger	*PRPH2*	sp	het	Exon 2	c.658C>T	p.Arg220Trp	[88]
137	m	25	nvp	54	np	red	red	Sanger	*PRPH2*	tbd	het	Exon 2	c.692C>G	p.Ser231*	novel
138	w	27	rva	33	np	red	red	Sanger	*PRPH2*	ad	het	Exon 1	c.310_313del	p.Ile104fs	[95]
139	m	51	rva	55	np	np	np	Sanger	*PRPH2*	sp	het	Exon 1	c.424C>T	p.Arg142Trp	[90,96]
140	m	50	met	50	np	red	red	NGS	*PRPH2*	sp	het	Exon 2	c.626T>A	p.Val209Asp	novel
141	w	46	met	48	np	red	na	Sanger	*PRPH2*	sp	het	Exon 1	c.441del	p.Gly148Alafs*5	[96,97]
142	w	46	rva	49	np	na	bor	Sanger	*PRPH2*	ad	het	Exon 1	c.514C>T	p.Arg172Trp	[83,89]
143	m	68	rva	75	np	np	np	Sanger	*PRPH2*	sp	het	Exon 1	c.513dup	p.Arg172Serfs*5	novel
144	m	50	met	53	nor	np	np	Sanger	*PRPH2*	sp	het	Exon 1	c.2T>C	p.Met1?	[84]
145	w	45	met, rva	56	np	nor	nor	Sanger	*PRPH2*	ad	het	Exon 2	c.664T>C	p.Cys222Arg	novel
146	m	50	met	60	bor	red	red	NGS	*PRPH2*	sp	het	Exon 2	c.749G>A	p.Cys250Tyr	novel
147	m	52	rva	52	bor	nor	nor	NGS	*PRPH2*	sp	het	Exon 1	c.441del	p.Gly148Alafs*5	[96,97]
148	w	88	rva	92	np	np	np	Sanger	*PRPH2*	sp	het	Exon 1	c.654_655del	p.Pro291Thrfs*81	novel
149	m	64	rva	80	red	red	red	Sanger	*PRPH2*	ad	het	Exon 1	c.178del	p.Val60Cysfs*5	novel
150	w	56	rva	65	np	red	red	NGS	*PRPH2*	ad	het	Exon 1	c.2T>C	p.Met1?	[84]
151	m	57	rva	77	np	red	red	NGS	*PRPH2*	sp	het	Exon 2	c.774C>G	p.Tyr258*	[89]
152	m	50	rva	53	bor	np	np	Sanger	*BEST1*	ad	het	Exon 7	c.728C>T	p.Ala243Val	[64]
153	m	chi	rva	16	red	np	np	Sanger	*BEST1*	ad	het	Exon 2	c.25G>A	p.Val9Met	[64]
154	w	48	rva	58	red	nor	nor	Sanger	*BEST1*	ad	het	Exon 2	c.17C>T	p.Thr6Ile	novel
155	m	49	nvp	55	red	nor	nor	Sanger	*BEST1*	sp	het	Exon 4	c.299T>G	p.Leu100Arg	[98]
156	m	72	rva	73	red	red	bor	Sanger	*BEST1*	sp	het	Exon 6	c.684C>G	p.Asp228Glu	novel
157	w	70	rva	78	red	red	bor	Sanger	*BEST1*	sp	het	Exon 8	c.934G>A	p.Asp312Asn	[64-66]
158	m	58	rva	64	red	np	np	Sanger	*BEST1*	ad	het	Exon 8	c.903T>G	p.Asp301Glu	[64,99]
159	w	69	rva	73	red	nor	nor	Sanger	*BEST1*	sp	het	Exon 2	c.62T>G	p.Leu21Arg	novel
160	w	55	met	58	red	np	np	Sanger	*BEST1*	sp	het	Exon 8	c.934G>A	p.Asp312Asn	[64-67]
161	m	47	rva	50	red	nor	nor	Sanger	*BEST1*	sp	het	Exon 4	c.428T>C	p.Val143Ala	novel
162	m	32	rva	48	red	nor	bor	Sanger	*BEST1*	ad	het	Exon 2	c.652C>T	p.Arg218Cys	[100]
163	m	50	rva	52	red	np	np	Sanger	*BEST1*	sp	het	Exon 7	c.728C>T	p.Ala243Val	[64]
164	m	chi	rva	49	red	red	red	Sanger	*BEST1*	sp	het	Exon 2	c.37C>T	p.Arg13Cys	[101]
165	m	17	rva	18	red	bor	red	Sanger	*BEST1*	sp	het	Exon 6	c.671T>C	p.Leu224Pro	[102]
166	w	40	pho	45	np	red	red	NGS	*GUCY2D*	ad	het	Exon 13	c.2492T>C	p.Leu831Pro	novel
167	m	23	rva	26	np	bor	red	NGS	*GUCY2D*	sp	het	Exon 13	c.2513G>A	p.Arg838His	[103]
168	m	50	rva	71	np	red	red	NGS	*GUCY2D*	sp	hom	Exon 2	c.380C>T	p.Pro127Leu	[104]***
169	w	25	rva, sco	46	np	red	red	Sanger	*GUCY2D*	ad	het	Exon 13	c.2512C>T	p.Arg838Cys	[83,105,106]
170	w	17	rva	35	np	na	na	Sanger	*GUCY2D*	ad	het	Exon 13	c.2512C>T	p.Arg838Cys	[83,105,106]
171	w	53	rva	61	np	nor	red	Sanger	*GUCY2D*	ad	het	Exon 13	c.2512C>T	p.Arg838Cys	[83,105,106]
172	w	7	rva	24	red	red	red	NGS	*CRX*	ad	het	Exon 4	c.590del	p.Pro197Argfs*22	novel
173	w	58	rva	58	np	nor	red	NGS	*CRX*	sp	het	Exon 3	c.159del	p.Glu53Aspfs*22	novel
174	w	50	pho	55	np	red	red	NGS	*CRX*	sp	het	Exon 4	c.434dup	p.Leu146Serfs*28	novel
175	w	chi	rva	26	np	red	ext	NGS	*CRX*	ad	het	Exon 4	c.663C>A	p.Tyr221*	novel
176	w	chi	rva	26	np	red	red	NGS	*GUCA1A*	ad	het	Exon 6	c.451C>T	p.Leu151Phe	[107]
177	m	47	rva	50	np	na	bor	NGS	*GUCA1A*	ad	het	Exon 6	c.526C>T	p.Leu176Phe	[108]
178	w	18	rva	61	np	red	ext	Sanger	*GUCA1A*	ad	het	Exon 6	c.451C>T	p.Leu151Phe	[107]
179	w	5	rva	11	np	red	red	NGS	*KIF11*	ad	het	Exon 14	c.1844_1846del	p.Ala615del	[109]
180	m	chi	rva	38	np	red	ext	NGS	*KIF11*	sp	het	Exon 8	c.808G>T	p.Glu270*	[109]

181	w	18	rva	43	np	red	red	NGS	*RP1L1*	ad	het	Exon 2	c.133C>T	p.Arg45Trp	[110]
182	w	46	rva	53	np	nor	red	NGS	*RP1L1*	sp	het	Exon 2	c.133C>T	p.Arg45Trp	[110]
183	w	65	met, rva	71	np	np	np	NGS	*C1QTNF5*	sp	het	Exon 15	c.489C>G	p.Ser163Arg	[111]
184	w	24	rva	24	np	bor	red	NGS	*JAG1*	sp	het	Exon 25	c.3164_3167del	p.Val1055Glufs*7	[112]
185	m	31	rva	33	np	nor	nor	NGS	*PROM1*	ad	het	Exon 10	c.1117C>T	p.Arg373Cys	[113]
186	m	58	rva	58	np	nor	nor	NGS	*IMPDH1*	ad	het	Exon 2	c.189A>G	p.Ser63Ser (splice site)	novel
187	w	77	rva	78	nor	np	np	NGS	*FBLN5*	sp	het	Exon 10	c.1093A>G	p.Ile365Val	novel
188	w	44	rva	48	bor	red	red	NGS	*SEMA4A*	sp	het	Exon 8	c.782dup	p.His261Glnfs*7	novel
**X-linked**															
189	m	40	rva	41	np	red	red	NGS	*RPGR*	sp	hemi	ORF15	c.3458A>C	p.(*1153Serext*38)	novel
**Mitochondrial**															
190	m	55	inf	55	np	na	na	NGS	*mtDNA*	sp	17%	MTTL1	m.3243A>G (17%)	n.a.	[114]
191	w	47	rva	49	np	red	red	NGS	*mtDNA*	sp	20%	MTTL1	m.3243A>G (20%)	n.a.	[114]

Age of onset: chi = childhood, ale = adolescence

First symptoms: nvp = night vision problems / dark adaption problems, pho = photophobia, met = metamorphopsia, rva = reduced visual acuity, inf = incidental finding, sco = scotoma, haz = haze

ERG, EOG: nor = normal, bor = borderline, red = reduced, ext = extinguished, np = not performed, na = not analyzable, eln = electronegative

Inheritance: ar = autosomal recessive, ad = autosomal dominant, sp = sporadic, het = heterozygous, hemi = hemizygous, pd = pseudodominant. For mitochondrial mutations percentages indicate the proportion of reads with mutation

Protein: p.c.u. = protein consequence unknown, n.a. = not applicable

* Reported in a patient without phenotype of Stargardt disease

** Regarding the patient's phenotype that is compatible with *ABCA4*-related disease, the *GUCY2D* variant most likely represents a rare neutral variant or, at the most, carriership for a recessive mutation

*** Reported in a patient with LCA

‡ In three patients the *ABCA4* variant c.5603A>T (p.Asn1868Ile; Exon 40) was found in combination with the heterozygous *ABCA4* variant c.5329A>T (p.Met1777Leu; Exon 38). However, only 4/10 functional prediction programs evaluated this novel variant as pathogenic and it was therefore excluded.

Supplementary Table 2

Identified novel mutations including allele frequencies and in silico prediction.

ID (#)	Gene	Exons/Introns (IVS)	Nucleotide	Protein	gnomAD exomes allel frequency (%)	Functional prediction	Conservation prediction
**Missense and splice variants**							
4	*ABCA4*	Exon 49	c.6746C>A	p.Ala2249Asp	n.a.	10/10	5/6
8	*ABCA4*	Exon 39	c.5509C>A	p.Pro1837Thr	0.00041	10/10	4/6
43	*ABCA4*	Exon 22	c.3243G>T	p.Lys1081Asn	n.a.	9/10	4/6
56	*ABCA4*	Exon 13	c.1807T>C	p.Tyr603His	n.a.	10/10	4/6
62	*ABCA4*	Exon 11	c.1523G>C	p.Arg508Pro	n.a.	7/10	4/6
77	*ABCA4*	Exon 30	c.4468T>C	p.Cys1490Arg	n.a.	10/10	5/6
78	*ABCA4*	Exon 35	c.4854G>T	p.Trp1618Cys	n.a.	10/10	5/6
81	*ABCA4*	Exon 6	c.694C>T	p.Leu232Phe	0.0057	7/10	4/6
86	*ABCA4*	Exon 15	c.2255G>A	p.Ser752Asn	n.a.	9/10	3/6
103	*BEST1*	Exon 9	c.956T>C	p.Leu319Pro	n.a.	9/10	5/6
109	*PROM1*	Exon 16	c.1853T>G	p.Leu618Arg	n.a.	9/10	4/6
111	*CERKL*	Exon 14	c.1651A>T	p.Ser551Cys	0.00041	6/10	4/6
113	*MERTK*	Exon 13<br>Exon 18	c.1801G>C<br>c.2360G>A	p.Val601Leu<br>p.Gly787Asp	0.00041<br>n.a.	8/9<br>9/9	3/6<br>5/6
119	*DRAM2*	Exon 3<br>Exon 5	c.47T>C<br>c.284G>T	p.Val16Ala<br>p.Gly95Val	0.00041<br>0.0028	6/10<br>10/10	5/6<br>5/6
121	*INPP5E*	Exon 2	c.844G>A	p.Gly282Arg	0.01	9/10	5/6
132	*PRPH2*<br>*(RIMS1)*	Exon 2	c.626T>A<br>c.4945T>A	p.Val209Asp<br>p.Ser1649Thr	n.a.<br>0.01	10/10<br>5/10	5/6<br>4/6
140	*PRPH2*	Exon 2	c.626T>A	p.Val209Asp	n.a.	10/10	5/6
145	*PRPH2*	Exon 2	c.664T>C	p.Cys222Arg	n.a.	10/10	5/6
146	*PRPH2*	Exon 2	c.749G>A	p.Cys250Tyr	n.a.	10/10	5/6
154	*BEST1*	Exon 2	c.17C>T	p.Thr6Ile	n.a.	8/9	5/6
156	*BEST1*	Exon 6	c.684C>G	p.Asp228Glu	0.00041	10/10	3/6
159	*BEST1*	Exon 2	c.62T>G	p.Leu21Arg	n.a.	9/9	5/6
161	*BEST1*	Exon 4	c.428T>C	p.Val143Ala	n.a.	8/10	5/6
166	*GUCY2D*	Exon 13	c.2492T>C	p.Leu831Pro	n.a.	9/10	5/6
186	*IMPDH1*	Exon 2	c.189A>G	p.Ser63Ser (splice site)	0.05	2/2	n.a
187	*FBLN5*	Exon 10	c.1093A>G	p.Ile365Val	0.0037	6/10	3/6
**Nonsense and frameshift variants, large deletions**							
15	*ABCA4*	Exon 13	c.1830T>A	p.Tyr610*	0.00041		
36	*ABCA4*	Exon 36	c.5189G>A	p.Trp1730*	0.00043		
37	*ABCA4*	Exons 12-13	deletion of Exons 12-13	putative loss of function	n.a.		
38	*ABCA4*	Exon 12	c.1584C>A	p.Tyr528*	n.a.		
39	*ABCA4*	Exon 36	c.5172G>A	p.Trp1724*	n.a.		
42	*ABCA4*	Exon 23	c.3468C>G	p.Tyr1156*	n.a.		
43	*ABCA4*	Exon 21	c.3098del	p.Lys1033Serfs*51	n.a.		
49	*ABCA4*	Exon 27	c.3871C>T	p.Gln1291*	0.00042		
61	*ABCA4*	Exon 46	c.6310dup	p.Gln2104Profs*31	n.a.		
65	*ABCA4*	Exon 6	c.741_744del	p.Asn247Lysfs*14	n.a.		
75	*ABCA4*	Exon 5	c.470T>A	p.Leu157*	n.a.		
77	*ABCA4*	Exon 30	c.4458delA	p.Ser1487Profs*39	n.a.		
100	*CDHR1*	Exon 14	c.1503_1507del	p.Gly502Leufs*32	n.a.		
106	*PROM1*	Exon 4<br>Exon 12	c.436C>T<br>c.1354dup	p.Arg146*<br>p.Tyr452Leufs*13	0.0012<br>n.a.		
107	*PROM1*	Exon 1	c.199C>T	p.Gln67*	n.a.		
108	*PROM1*	Exon 13	c.1354dup	p.Tyr452Leufs*13	n.a.		
112	*CERKL*	Exon 1<br>Exon 2	c.197_200dup<br>whole-exon deletion	p.Leu68Serfs*15<br>putative loss of function	n.a.<br>n.a.		
114	*MERTK*	Exons 2-19<br>Exon 2	deletion of Exons 2-19<br>c.369C>G	putative loss of function<br>p.Tyr123*	n.a.<br>n.a.		
122	*FAM161A*	Exon 3	c.971del	p.Pro324Hisfs*5	n.a.		
127	*PRPH2*	Exon 1	c.571G>T	p.Glu191*	n.a.		
129	*PRPH2*	Exon 2	c.612C>G	p.Tyr204*	n.a.		
134	*PRPH2*	Exon 2	c.771C>G	p.Tyr257*	n.a.		
137	*PRPH2*	Exon 2	c.692C>G	p.Ser231*	n.a.		
143	*PRPH2*	Exon 1	c.513dup	p.Arg172Serfs*5	n.a.		
148	*PRPH2*	Exon 1	c.654_655del	p.Pro291Thrfs*81	n.a.		
149	*PRPH2*	Exon 1	c.178del	p.Val60Cysfs*5	n.a.		
172	*CRX*	Exon 4	c.590del	p.Pro197Argfs*22	n.a.		
173	*CRX*	Exon 3	c.159del	p.Glu53Aspfs*22	n.a.		
174	*CRX*	Exon 4	c.434dup	p.Leu146Serfs*28	n.a.		
175	*CRX*	Exon 4	c.663C>A	p.Tyr221*	n.a.		
188	*SEMA4A*	Exon 8	c.782dup	p.His261Glnfs*7	n.a.		
189	*RPGR*	ORF15	c.3458A>C	p.(*1153Serext*38)	n.a.		

Functional predictions: Functional predictions by SIFT, PolyPhen2, MutationTaster, MutationAssessor, FATHMM, LRT, VEST, CADD, PROVEAN and DANN; Functional predictions for spice sites: AdaBoost, RF

Conservation predictions: Assessment of conservation by PhyloP, GERP++, PhastCons, SiPhy, Grantham Distance and BLOSUM62.

n.a. = not available

Supplementary Table 3

Pathways and cellular compartments involved in the retinal pathophysiology of this study's retinal diseases.

Gene	Functional category
INPP5E	cilium function
POC1B	
FAM161A	
NPHP1	
RPGR	
RP1L1	
PRPH2	structure and morphogenesis
CDHR1	
PROM1	
GUCY2D	phototransduction
GUCA1A	
ABCA4	visual cycle
RDH5	
CRB1	cell adhesion/structure
CDH3	
C1QTNF5	extracellular matrix
FBLN5	
KIF11	cell differentiation/mitosis
JAG1	
SEMA4A	unknown function
CERKL	
IMPDH1	cell cycle
CRX	transcription
MERTK	phagocytosis
DRAM2	apoptosis
BEST1	transmembrane channel

Supplementary Table 4

Patients with only one mutation in a gene causing recessive retinopathy in combination with a phenotype usually associated with mutations in the respective gene.

Gene	Genotype	Exons/ Introns (IVS)	Nucleotide	Protein	reference
ABCA4	Heterozygous	Exon 42	c.5882G>A	p.Gly1961Glu	[4-6]
ABCA4	Heterozygous	Exon 12<br>Exon 21	c.1622T>C<br>c.3113C>T	p.Leu541Pro<br>p.Ala1038Val	[12,13]<br>[12,13]
ABCA4	Heterozygous	Exon 48	c.6601_6602del	p.Arg2201Alafs*49	novel
ABCA4	Heterozygous	Exon 1	c.66G>A	splice site	novel
ABCA4	Heterozygous	Exon 6	c.768G>T	splice site	[1,26]
CDH3	Heterozygous	Exon 9	c.1028del	p.Gly343Alafs*7	novel
CERKL ‡	Heterozygous	Exon 6	c.847C>T	p.Arg283*	[72]

‡ The shown variant was found in combination with a novel heterozygous splice site variant in *CERKL* (c.1212-3T>A; Intron 9). However, none of the applied functional prediction programs for spice sites (AdaBoost, RF) evaluated this novel variant as pathogenic and therefore the patient was grouped in the unsolved cohort.

References

1 Maugeri, A. *et al.* The 2588G-->C mutation in the ABCR gene is a mild frequent founder mutation in the Western European population and allows the classification of ABCR mutations in patients with Stargardt disease. *Am J Hum Genet* **64**, 1024-1035, doi:AJHG980765 (1999).

2 Bertelsen, M. *et al.* Generalized choriocapillaris dystrophy, a distinct phenotype in the spectrum of ABCA4-associated retinopathies. *Invest Ophthalmol Vis Sci* **55**, 2766-2776, doi:10.1167/iovs.13-13391 (2014).

3 Duncker, T. *et al.* Quantitative fundus autofluorescence distinguishes ABCA4-associated and non-ABCA4-associated bull's-eye maculopathy. *Ophthalmology* **122**, 345-355, doi:10.1016/j.ophtha.2014.08.017 (2015).

4 Rivera, A. *et al.* A comprehensive survey of sequence variation in the ABCA4 (ABCR) gene in Stargardt disease and age-related macular degeneration. *Am J Hum Genet* **67**, 800-813, doi:10.1086/303090 (2000).

5 Sun, H., Smallwood, P. M. & Nathans, J. Biochemical defects in ABCR protein variants associated with human retinopathies. *Nat Genet* **26**, 242-246, doi:10.1038/79994 (2000).

6 Cella, W. *et al.* G1961E mutant allele in the Stargardt disease gene ABCA4 causes bull's eye maculopathy. *Exp Eye Res* **89**, 16-24, doi:10.1016/j.exer.2009.02.001 (2009).

7 Rozet, J. M. *et al.* Spectrum of ABCR gene mutations in autosomal recessive macular dystrophies. *Eur J Hum Genet* **6**, 291-295, doi:10.1038/sj.ejhg.5200221 (1998).

8 Sabirzhanova, I. *et al.* Rescuing Trafficking Mutants of the ATP-binding Cassette Protein, ABCA4, with Small Molecule Correctors as a Treatment for Stargardt Eye Disease. *J Biol Chem* **290**, 19743-19755, doi:10.1074/jbc.M115.647685 (2015).

9 Riveiro-Alvarez, R. *et al.* Outcome of ABCA4 disease-associated alleles in autosomal recessive retinal dystrophies: retrospective analysis in 420 Spanish families. *Ophthalmology* **120**, 2332-2337, doi:10.1016/j.ophtha.2013.04.002 (2013).

10 Lewis, R. A. *et al.* Genotype/Phenotype analysis of a photoreceptor-specific ATP-binding cassette transporter gene, ABCR, in Stargardt disease. *Am J Hum Genet* **64**, 422-434, doi:10.1086/302251 (1999).

11 Thiadens, A. A. *et al.* Clinical course, genetic etiology, and visual outcome in cone and cone-rod dystrophy. *Ophthalmology* **119**, 819-826, doi:10.1016/j.ophtha.2011.10.011 (2012).

12 Webster, A. R. *et al.* An analysis of allelic variation in the ABCA4 gene. *Invest Ophthalmol Vis Sci* **42**, 1179-1189 (2001).

13 Aguirre-Lamban, J. *et al.* Further associations between mutations and polymorphisms in the ABCA4 gene: clinical implication of allelic variants and their role as protector/risk factors. *Invest Ophthalmol Vis Sci* **52**, 6206-6212, doi:10.1167/iovs.10-5743 (2011).

14 Fujinami, K. *et al.* Clinical and molecular analysis of Stargardt disease with preserved foveal structure and function. *Am J Ophthalmol* **156**, 487-501 e481, doi:10.1016/j.ajo.2013.05.003 (2013).

15 Grassmann, F. *et al.* Common synonymous variants in ABCA4 are protective for chloroquine induced maculopathy (toxic maculopathy). *BMC Ophthalmol* **15**, 18, doi:10.1186/s12886-015-0008-0 (2015).

16 Zernant, J. *et al.* Analysis of the ABCA4 gene by next-generation sequencing. *Invest Ophthalmol Vis Sci* **52**, 8479-8487, doi:10.1167/iovs.11-8182 (2011).

17 Allikmets, R. A photoreceptor cell-specific ATP-binding transporter gene (ABCR) is mutated in recessive Stargardt macular dystrophy. *Nat Genet* **17**, 122, doi:10.1038/ng0997-122a (1997).

18 Gerber, S. *et al.* Complete exon-intron structure of the retina-specific ATP binding transporter gene (ABCR) allows the identification of novel mutations underlying Stargardt disease. *Genomics* **48**, 139-142, doi:10.1006/geno.1997.5164 (1998).

19 Suarez, T., Biswas, S. B. & Biswas, E. E. Biochemical defects in retina-specific human ATP binding cassette transporter nucleotide binding domain 1 mutants associated with macular degeneration. *J Biol Chem* **277**, 21759-21767, doi:10.1074/jbc.M202053200 (2002).

20 Aguirre-Lamban, J. *et al.* Novel human pathological mutations. Gene symbol: ABCA4. Disease: Stargardt disease. *Hum Genet* **127**, 119 (2010).
21 Michaelides, M. *et al.* ABCA4 mutations and discordant ABCA4 alleles in patients and siblings with bull's-eye maculopathy. *Br J Ophthalmol* **91**, 1650-1655, doi:10.1136/bjo.2007.118356 (2007).
22 Cornelis, S. S. *et al.* In Silico Functional Meta-Analysis of 5,962 ABCA4 Variants in 3,928 Retinal Dystrophy Cases. *Hum Mutat* **38**, 400-408, doi:10.1002/humu.23165 (2017).
23 Cideciyan, A. V. *et al.* ABCA4 disease progression and a proposed strategy for gene therapy. *Hum Mol Genet* **18**, 931-941, doi:10.1093/hmg/ddn421 (2009).
24 Muller, P. L. *et al.* Monoallelic ABCA4 Mutations Appear Insufficient to Cause Retinopathy: A Quantitative Autofluorescence Study. *Invest Ophthalmol Vis Sci* **56**, 8179-8186, doi:10.1167/iovs.15-17629 (2015).
25 Tiwari, A. *et al.* Next generation sequencing based identification of disease-associated mutations in Swiss patients with retinal dystrophies. *Sci Rep* **6**, 28755, doi:10.1038/srep28755 (2016).
26 Klevering, B. J. *et al.* Three families displaying the combination of Stargardt's disease with cone-rod dystrophy or retinitis pigmentosa. *Ophthalmology* **111**, 546-553, doi:10.1016/j.ophtha.2003.06.010 (2004).
27 Jaakson, K. *et al.* Genotyping microarray (gene chip) for the ABCR (ABCA4) gene. *Hum Mutat* **22**, 395-403, doi:10.1002/humu.10263 (2003).
28 Downs, K. *et al.* Molecular testing for hereditary retinal disease as part of clinical care. *Arch Ophthalmol* **125**, 252-258, doi:10.1001/archopht.125.2.252 (2007).
29 Battu, R. *et al.* Identification of Novel Mutations in ABCA4 Gene: Clinical and Genetic Analysis of Indian Patients with Stargardt Disease. *Biomed Res Int* **2015**, 940864, doi:10.1155/2015/940864 (2015).
30 Schulz, H. L. *et al.* Mutation Spectrum of the ABCA4 Gene in 335 Stargardt Disease Patients From a Multicenter German Cohort-Impact of Selected Deep Intronic Variants and Common SNPs. *Invest Ophthalmol Vis Sci* **58**, 394-403, doi:10.1167/iovs.16-19936 (2017).
31 Fishman, G. A. *et al.* Variation of clinical expression in patients with Stargardt dystrophy and sequence variations in the ABCR gene. *Arch Ophthalmol* **117**, 504-510 (1999).
32 Allikmets, R. *et al.* Mutation of the Stargardt disease gene (ABCR) in age-related macular degeneration. *Science* **277**, 1805-1807 (1997).
33 Simonelli, F. *et al.* New ABCR mutations and clinical phenotype in Italian patients with Stargardt disease. *Invest Ophthalmol Vis Sci* **41**, 892-897 (2000).
34 Shroyer, N. F., Lewis, R. A., Yatsenko, A. N. & Lupski, J. R. Null missense ABCR (ABCA4) mutations in a family with stargardt disease and retinitis pigmentosa. *Invest Ophthalmol Vis Sci* **42**, 2757-2761 (2001).
35 Klevering, B. J., Deutman, A. F., Maugeri, A., Cremers, F. P. & Hoyng, C. B. The spectrum of retinal phenotypes caused by mutations in the ABCA4 gene. *Graefes Arch Clin Exp Ophthalmol* **243**, 90-100, doi:10.1007/s00417-004-1079-4 (2005).
36 Cremers, F. P. *et al.* Autosomal recessive retinitis pigmentosa and cone-rod dystrophy caused by splice site mutations in the Stargardt's disease gene ABCR. *Hum Mol Genet* **7**, 355-362 (1998).
37 Klevering, B. J. *et al.* Phenotypic variations in a family with retinal dystrophy as result of different mutations in the ABCR gene. *Br J Ophthalmol* **83**, 914-918 (1999).
38 Briggs, C. E. *et al.* Mutations in ABCR (ABCA4) in patients with Stargardt macular degeneration or cone-rod degeneration. *Invest Ophthalmol Vis Sci* **42**, 2229-2236 (2001).
39 Allikmets, R. *et al.* A photoreceptor cell-specific ATP-binding transporter gene (ABCR) is mutated in recessive Stargardt macular dystrophy. *Nat Genet* **15**, 236-246, doi:10.1038/ng0397-236 (1997).
40 Stenirri, S. *et al.* Molecular scanning of the ABCA4 gene in Spanish patients with retinitis pigmentosa and Stargardt disease: identification of novel mutations. *Eur J Ophthalmol* **17**, 749-754 (2007).

41 Bravo-Gil, N. *et al.* Improving the management of Inherited Retinal Dystrophies by targeted sequencing of a population-specific gene panel. *Sci Rep* **6**, 23910, doi:10.1038/srep23910 (2016).
42 Alapati, A. *et al.* Molecular diagnostic testing by eyeGENE: analysis of patients with hereditary retinal dystrophy phenotypes involving central vision loss. *Invest Ophthalmol Vis Sci* **55**, 5510-5521, doi:10.1167/iovs.14-14359 (2014).
43 Ernest, P. J., Boon, C. J., Klevering, B. J., Hoefsloot, L. H. & Hoyng, C. B. Outcome of ABCA4 microarray screening in routine clinical practice. *Mol Vis* **15**, 2841-2847 (2009).
44 Jiang, F. *et al.* Screening of ABCA4 Gene in a Chinese Cohort With Stargardt Disease or Cone-Rod Dystrophy With a Report on 85 Novel Mutations. *Invest Ophthalmol Vis Sci* **57**, 145-152, doi:10.1167/iovs.15-18190 (2016).
45 Rosenberg, T., Klie, F., Garred, P. & Schwartz, M. N965S is a common ABCA4 variant in Stargardt-related retinopathies in the Danish population. *Mol Vis* **13**, 1962-1969 (2007).
46 Biswas-Fiss, E. E. Functional analysis of genetic mutations in nucleotide binding domain 2 of the human retina specific ABC transporter. *Biochemistry* **42**, 10683-10696, doi:10.1021/bi034481l (2003).
47 Corton, M. *et al.* High frequency of CRB1 mutations as cause of Early-Onset Retinal Dystrophies in the Spanish population. *Orphanet J Rare Dis* **8**, 20, doi:10.1186/1750-1172-8-20 (2013).
48 Poloschek, C. M. *et al.* ABCA4 and ROM1: implications for modification of the PRPH2-associated macular dystrophy phenotype. *Invest Ophthalmol Vis Sci* **51**, 4253-4265, doi:10.1167/iovs.09-4655 (2010).
49 Souied, E. H. *et al.* A novel ABCR nonsense mutation responsible for late-onset fundus flavimaculatus. *Invest Ophthalmol Vis Sci* **40**, 2740-2744 (1999).
50 Roberts, L. J., Nossek, C. A., Greenberg, L. J. & Ramesar, R. S. Stargardt macular dystrophy: common ABCA4 mutations in South Africa--establishment of a rapid genetic test and relating risk to patients. *Mol Vis* **18**, 280-289 (2012).
51 Perrault, I. *et al.* Spectrum of retGC1 mutations in Leber's congenital amaurosis. *Eur J Hum Genet* **8**, 578-582, doi:10.1038/sj.ejhg.5200503 (2000).
52 Littink, K. W. *et al.* Homozygosity mapping in patients with cone-rod dystrophy: novel mutations and clinical characterizations. *Invest Ophthalmol Vis Sci* **51**, 5943-5951, doi:10.1167/iovs.10-5797 (2010).
53 Oldani, M. *et al.* Clinical and molecular genetic study of 12 Italian families with autosomal recessive Stargardt disease. *Genet Mol Res* **11**, 4342-4350, doi:10.4238/2012.October.9.3 (2012).
54 Downes, S. M. *et al.* Detection rate of pathogenic mutations in ABCA4 using direct sequencing: clinical and research implications. *Arch Ophthalmol* **130**, 1486-1490, doi:10.1001/archophthalmol.2012.1697 (2012).
55 Huang, X. F. *et al.* Genotype-phenotype correlation and mutation spectrum in a large cohort of patients with inherited retinal dystrophy revealed by next-generation sequencing. *Genet Med* **17**, 271-278, doi:10.1038/gim.2014.138 (2015).
56 Stenirri, S. *et al.* Denaturing HPLC profiling of the ABCA4 gene for reliable detection of allelic variations. *Clin Chem* **50**, 1336-1343, doi:10.1373/clinchem.2004.033241 (2004).
57 Klevering, B. J. *et al.* Phenotypic spectrum of autosomal recessive cone-rod dystrophies caused by mutations in the ABCA4 (ABCR) gene. *Invest Ophthalmol Vis Sci* **43**, 1980-1985 (2002).
58 Lambertus, S. *et al.* Early-onset stargardt disease: phenotypic and genotypic characteristics. *Ophthalmology* **122**, 335-344, doi:10.1016/j.ophtha.2014.08.032 (2015).
59 Fujinami, K. *et al.* Clinical and molecular characteristics of childhood-onset Stargardt disease. *Ophthalmology* **122**, 326-334, doi:10.1016/j.ophtha.2014.08.012 (2015).
60 Braun, T. A. *et al.* Non-exomic and synonymous variants in ABCA4 are an important cause of Stargardt disease. *Hum Mol Genet* **22**, 5136-5145, doi:10.1093/hmg/ddt367 (2013).

61 Duno, M., Schwartz, M., Larsen, P. L. & Rosenberg, T. Phenotypic and genetic spectrum of Danish patients with ABCA4-related retinopathy. *Ophthalmic Genet* **33**, 225-231, doi:10.3109/13816810.2011.643441 (2012).
62 Baum, L. *et al.* ABCA4 sequence variants in Chinese patients with age-related macular degeneration or Stargardt's disease. *Ophthalmologica* **217**, 111-114, doi:68553 (2003).
63 Stingl, K. *et al.* CDHR1 mutations in retinal dystrophies. *Sci Rep* **7**, 6992, doi:10.1038/s41598-017-07117-8 (2017).
64 Kramer, F. *et al.* Mutations in the VMD2 gene are associated with juvenile-onset vitelliform macular dystrophy (Best disease) and adult vitelliform macular dystrophy but not age-related macular degeneration. *Eur J Hum Genet* **8**, 286-292, doi:10.1038/sj.ejhg.5200447 (2000).
65 Boon, C. J. *et al.* Autosomal recessive bestrophinopathy: differential diagnosis and treatment options. *Ophthalmology* **120**, 809-820, doi:10.1016/j.ophtha.2012.09.057 (2013).
66 Johnson, A. A. *et al.* Disease-causing mutations associated with four bestrophinopathies exhibit disparate effects on the localization, but not the oligomerization, of Bestrophin-1. *Exp Eye Res* **121**, 74-85, doi:10.1016/j.exer.2014.02.006 (2014).
67 Burgess, R. *et al.* Biallelic mutation of BEST1 causes a distinct retinopathy in humans. *Am J Hum Genet* **82**, 19-31, doi:10.1016/j.ajhg.2007.08.004 (2008).
68 Johnson, A. A. *et al.* Autosomal Recessive Bestrophinopathy Is Not Associated With the Loss of Bestrophin-1 Anion Channel Function in a Patient With a Novel BEST1 Mutation. *Invest Ophthalmol Vis Sci* **56**, 4619-4630, doi:10.1167/iovs.15-16910 (2015).
69 Lee, C. S. *et al.* A Novel BEST1 Mutation in Autosomal Recessive Bestrophinopathy. *Invest Ophthalmol Vis Sci* **56**, 8141-8150, doi:10.1167/iovs.15-18168 (2015).
70 Glockle, N. *et al.* Panel-based next generation sequencing as a reliable and efficient technique to detect mutations in unselected patients with retinal dystrophies. *Eur J Hum Genet* **22**, 99-104, doi:10.1038/ejhg.2013.72 (2014).
71 Crowley, C. *et al.* Autosomal recessive bestrophinopathy associated with angle-closure glaucoma. *Doc Ophthalmol* **129**, 57-63, doi:10.1007/s10633-014-9444-z (2014).
72 Tuson, M., Marfany, G. & Gonzalez-Duarte, R. Mutation of CERKL, a novel human ceramide kinase gene, causes autosomal recessive retinitis pigmentosa (RP26). *Am J Hum Genet* **74**, 128-138, doi:10.1086/381055 (2004).
73 Caridi, G. *et al.* Renal-retinal syndromes: association of retinal anomalies and recessive nephronophthisis in patients with homozygous deletion of the NPH1 locus. *Am J Kidney Dis* **32**, 1059-1062, doi:S0272638698003643 (1998).
74 Parisi, M. A. *et al.* The NPHP1 gene deletion associated with juvenile nephronophthisis is present in a subset of individuals with Joubert syndrome. *Am J Hum Genet* **75**, 82-91, doi:10.1086/421846 (2004).
75 Indelman, M. *et al.* A missense mutation in CDH3, encoding P-cadherin, causes hypotrichosis with juvenile macular dystrophy. *J Invest Dermatol* **119**, 1210-1213, doi:10.1046/j.1523-1747.2002.19528.x (2002).
76 Vallespin, E. *et al.* Gene symbol: CRB1. Disease: Leber congenital amaurosis. Accession #Hd0510. *Hum Genet* **118**, 774 (2006).
77 Cideciyan, A. V. *et al.* Rod and cone visual cycle consequences of a null mutation in the 11-cis-retinol dehydrogenase gene in man. *Vis Neurosci* **17**, 667-678 (2000).
78 Ruther, K. *et al.* [Clinical and genetic findings in a patient with fundus albipunctatus]. *Ophthalmologe* **101**, 177-185, doi:10.1007/s00347-003-0895-y (2004).
79 Roosing, S. *et al.* Disruption of the basal body protein POC1B results in autosomal-recessive cone-rod dystrophy. *Am J Hum Genet* **95**, 131-142, doi:10.1016/j.ajhg.2014.06.012 (2014).
80 Durlu, Y. K., Koroglu, C. & Tolun, A. Novel recessive cone-rod dystrophy caused by POC1B mutation. *JAMA Ophthalmol* **132**, 1185-1191, doi:10.1001/jamaophthalmol.2014.1658 (2014).
81 Travaglini, L. *et al.* Phenotypic spectrum and prevalence of INPP5E mutations in Joubert syndrome and related disorders. *Eur J Hum Genet* **21**, 1074-1078, doi:10.1038/ejhg.2012.305 (2013).

82 Gruning, G. *et al.* Mutations in the human peripherin/RDS gene associated with autosomal dominant retinitis pigmentosa. *Hum Mutat* **3**, 321-323, doi:10.1002/humu.1380030326 (1994).

83 Kohl, S. *et al.* Genes and mutations in autosomal dominant cone and cone-rod dystrophy. *Adv Exp Med Biol* **723**, 337-343, doi:10.1007/978-1-4614-0631-0_44 (2012).

84 Felbor, U., Schilling, H. & Weber, B. H. Adult vitelliform macular dystrophy is frequently associated with mutations in the peripherin/RDS gene. *Hum Mutat* **10**, 301-309, doi:10.1002/(SICI)1098-1004(1997)10:4<301::AID-HUMU6>3.0.CO;2-J (1997).

85 Kohl, S. *et al.* RDS/peripherin gene mutations are frequent causes of central retinal dystrophies. *J Med Genet* **34**, 620-626 (1997).

86 Manes, G. *et al.* High prevalence of PRPH2 in autosomal dominant retinitis pigmentosa in france and characterization of biochemical and clinical features. *Am J Ophthalmol* **159**, 302-314, doi:10.1016/j.ajo.2014.10.033 (2015).

87 Trujillo Tiebas, M. J., Gimenez Pardo, A., Garcia Sandoval, B. & Ayuso Garcia, C. [Phenotypic variation in a family affected by autosomal dominant retinal dystrophy caused by the Gly208Asp mutation in the RDS peripherin gene]. *Med Clin (Barc)* **118**, 716 (2002).

88 Payne, A. M., Downes, S. M., Bessant, D. A., Bird, A. C. & Bhattacharya, S. S. Founder effect, seen in the British population, of the 172 peripherin/RDS mutation-and further refinement of genetic positioning of the peripherin/RDS gene. *Am J Hum Genet* **62**, 192-195, doi:10.1086/301679 (1998).

89 Wells, J. *et al.* Mutations in the human retinal degeneration slow (RDS) gene can cause either retinitis pigmentosa or macular dystrophy. *Nat Genet* **3**, 213-218, doi:10.1038/ng0393-213 (1993).

90 Smailhodzic, D. *et al.* Central areolar choroidal dystrophy (CACD) and age-related macular degeneration (AMD): differentiating characteristics in multimodal imaging. *Invest Ophthalmol Vis Sci* **52**, 8908-8918, doi:10.1167/iovs.11-7926 (2011).

91 Meins, M. *et al.* Heterozygous 'null allele' mutation in the human peripherin/RDS gene. *Hum Mol Genet* **2**, 2181-2182 (1993).

92 Abu-Safieh, L. *et al.* Autozygome-guided exome sequencing in retinal dystrophy patients reveals pathogenetic mutations and novel candidate disease genes. *Genome Res* **23**, 236-247, doi:10.1101/gr.144105.112 (2013).

93 Leroy, B. P., Kailasanathan, A., De Laey, J. J., Black, G. C. & Manson, F. D. Intrafamilial phenotypic variability in families with RDS mutations: exclusion of ROM1 as a genetic modifier for those with retinitis pigmentosa. *Br J Ophthalmol* **91**, 89-93, doi:10.1136/bjo.2006.101915 (2007).

94 Hoyng, C. B. *et al.* Autosomal dominant central areolar choroidal dystrophy caused by a mutation in codon 142 in the peripherin/RDS gene. *Am J Ophthalmol* **121**, 623-629 (1996).

95 Duncker, T. *et al.* Quantitative Fundus Autofluorescence and Optical Coherence Tomography in PRPH2/RDS- and ABCA4-Associated Disease Exhibiting Phenotypic Overlap. *Invest Ophthalmol Vis Sci* **56**, 3159-3170, doi:10.1167/iovs.14-16343 (2015).

96 Neveling, K. *et al.* Next-generation genetic testing for retinitis pigmentosa. *Hum Mutat* **33**, 963-972, doi:10.1002/humu.22045 (2012).

97 Trujillo, M. J. *et al.* Three novel RDS-peripherin mutations (689delT, 857del17, G208D) in Spanish families affected with autosomal dominant retinal degenerations. Mutations in brief no. 147. Online. *Hum Mutat* **12**, 70, doi:10.1002/(SICI)1098-1004(1998)12:1<70::AID-HUMU13>3.0.CO;2-G (1998).

98 Kramer, F., Mohr, N., Kellner, U., Rudolph, G. & Weber, B. H. Ten novel mutations in VMD2 associated with Best macular dystrophy (BMD). *Hum Mutat* **22**, 418, doi:10.1002/humu.9189 (2003).

99 Caldwell, G. M. *et al.* Bestrophin gene mutations in patients with Best vitelliform macular dystrophy. *Genomics* **58**, 98-101, doi:10.1006/geno.1999.5808 (1999).

100 Kay, D. B. *et al.* Outer retinal structure in best vitelliform macular dystrophy. *JAMA Ophthalmol* **131**, 1207-1215, doi:10.1001/jamaophthalmol.2013.387 (2013).

101 Kinnick, T. R. *et al.* Autosomal recessive vitelliform macular dystrophy in a large cohort of vitelliform macular dystrophy patients. *Retina* **31**, 581-595, doi:10.1097/IAE.0b013e318203ee60 (2011).

102 Lotery, A. J. *et al.* Allelic variation in the VMD2 gene in best disease and age-related macular degeneration. *Invest Ophthalmol Vis Sci* **41**, 1291-1296 (2000).

103 Payne, A. M. *et al.* Clustering and frequency of mutations in the retinal guanylate cyclase (GUCY2D) gene in patients with dominant cone-rod dystrophies. *J Med Genet* **38**, 611-614 (2001).

104 Astuti, G. D. *et al.* Comprehensive genotyping reveals RPE65 as the most frequently mutated gene in Leber congenital amaurosis in Denmark. *Eur J Hum Genet* **24**, 1071-1079, doi:10.1038/ejhg.2015.241 (2016).

105 Kelsell, R. E. *et al.* Mutations in the retinal guanylate cyclase (RETGC-1) gene in dominant cone-rod dystrophy. *Hum Mol Genet* **7**, 1179-1184 (1998).

106 Wilkie, S. E. *et al.* Functional characterization of missense mutations at codon 838 in retinal guanylate cyclase correlates with disease severity in patients with autosomal dominant cone-rod dystrophy. *Hum Mol Genet* **9**, 3065-3073 (2000).

107 Sokal, I. *et al.* A novel GCAP1 missense mutation (L151F) in a large family with autosomal dominant cone-rod dystrophy (adCORD). *Invest Ophthalmol Vis Sci* **46**, 1124-1132, doi:10.1167/iovs.04-1431 (2005).

108 Weisschuh, N. *et al.* Mutation Detection in Patients with Retinal Dystrophies Using Targeted Next Generation Sequencing. *PLoS One* **11**, e0145951, doi:10.1371/journal.pone.0145951 (2016).

109 Birtel, J. *et al.* Novel Insights Into the Phenotypical Spectrum of KIF11-Associated Retinopathy, Including a New Form of Retinal Ciliopathy. *Invest Ophthalmol Vis Sci* **58**, 3950-3959, doi:10.1167/iovs.17-21679 (2017).

110 Akahori, M. *et al.* Dominant mutations in RP1L1 are responsible for occult macular dystrophy. *Am J Hum Genet* **87**, 424-429, doi:10.1016/j.ajhg.2010.08.009 (2010).

111 Hayward, C. *et al.* Mutation in a short-chain collagen gene, CTRP5, results in extracellular deposit formation in late-onset retinal degeneration: a genetic model for age-related macular degeneration. *Hum Mol Genet* **12**, 2657-2667, doi:10.1093/hmg/ddg289 (2003).

112 Crosnier, C. *et al.* Mutations in JAGGED1 gene are predominantly sporadic in Alagille syndrome. *Gastroenterology* **116**, 1141-1148 (1999).

113 Michaelides, M. *et al.* The PROM1 mutation p.R373C causes an autosomal dominant bull's eye maculopathy associated with rod, rod-cone, and macular dystrophy. *Invest Ophthalmol Vis Sci* **51**, 4771-4780, doi:10.1167/iovs.09-4561 (2010).

114 de Laat, P., Smeitink, J. A., Janssen, M. C., Keunen, J. E. & Boon, C. J. Mitochondrial retinal dystrophy associated with the m.3243A>G mutation. *Ophthalmology* **120**, 2684-2696, doi:10.1016/j.ophtha.2013.05.013 (2013).
